# Cardiomyocyte Regulation of Systemic Lipid Metabolism by the Apolipoprotein B-Containing Lipoproteins in *Drosophila*

**DOI:** 10.1371/journal.pgen.1006555

**Published:** 2017-01-17

**Authors:** Sunji Lee, Hong Bao, Zachary Ishikawa, Weidong Wang, Hui-Ying Lim

**Affiliations:** 1 Aging and Metabolism Research Program, Oklahoma Medical Research Foundation, Oklahoma City, Oklahoma, United States of America; 2 Department of Physiology, University of Oklahoma Health Science Center, Oklahoma City, Oklahoma, United States of America; 3 Department of Medicine, Section of Endocrinology, University of Oklahoma Health Sciences Center, Oklahoma City, Oklahoma, United States of America; Children's National Health System, UNITED STATES

## Abstract

The heart has emerged as an important organ in the regulation of systemic lipid homeostasis; however, the underlying mechanism remains poorly understood. Here, we show that *Drosophila* cardiomyocytes regulate systemic lipid metabolism by producing apolipoprotein B-containing lipoproteins (apoB-lipoproteins), essential lipid carriers that are so far known to be generated only in the fat body. In a *Drosophila* genetic screen, we discovered that when haplo-insufficient, *microsomal triglyceride transfer protein* (*mtp*), required for the biosynthesis of apoB-lipoproteins, suppressed the development of diet-induced obesity. Tissue-specific inhibition of Mtp revealed that whereas knockdown of *mtp* only in the fat body decreases systemic triglyceride (TG) content on normal food diet (NFD) as expected, knockdown of *mtp* only in the cardiomyocytes also equally decreases systemic TG content on NFD, suggesting that the cardiomyocyte- and fat body-derived apoB-lipoproteins serve similarly important roles in regulating whole-body lipid metabolism. Unexpectedly, on high fat diet (HFD), knockdown of *mtp* in the cardiomyocytes, but not in fat body, protects against the gain in systemic TG levels. We further showed that inhibition of the *Drosophila* apoB homologue, apolipophorin or apoLpp, another gene essential for apoB-lipoprotein biosynthesis, affects systemic TG levels similarly to that of Mtp inhibition in the cardiomyocytes on NFD or HFD. Finally, we determined that HFD differentially alters Mtp and apoLpp expression in the cardiomyocytes versus the fat body, culminating in higher Mtp and apoLpp levels in the cardiomyocytes than in fat body and possibly underlying the predominant role of cardiomyocyte-derived apoB-lipoproteins in lipid metabolic regulation. Our findings reveal a novel and significant function of heart-mediated apoB-lipoproteins in controlling lipid homeostasis.

## Author Summary

The heart is increasingly recognized to serve an important role in the regulation of whole-body lipid homeostasis; however, the underlying mechanisms remained poorly understood. Here, our study in *Drosophila* reveals that cardiomyocytes regulate systemic lipid metabolism by producing apolipoprotein B-containing lipoproteins (apoB-lipoproteins), essential lipid carriers that are so far known to be generated only in the fat body (insect liver and adipose tissue). We found that apoB-lipoproteins generated by the *Drosophila* cardiomyocytes serve an equally significant role as their fat body-derived counterparts in maintaining systemic lipid homeostasis on normal food diet. Importantly, on high fat diet (HFD), the cardiomyocyte-derived apoB-lipoproteins are the major determinants of whole-body lipid metabolism, a role which could be attributed to the HFD-induced up-regulation of apoB-lipoprotein biosynthesis genes in the cardiomyocytes and their down-regulation in the fat body. Taken together, our results reveal that apoB-lipoproteins are new players in mediating the heart control of lipid metabolism, and provide first evidence supporting the notion that HFD-induced differential regulation of apoB-lipoprotein biosynthesis genes could alter the input of different tissue-derived apoB-lipoproteins in systemic lipid metabolic control.

## Introduction

Obesity, a condition caused by a mismatch between energy consumption and utilization, is a significant risk factor for the development of Type II diabetes, hypertension and coronary heart disease [1–3]. The aetiology of obesity is multifactorial, but it is widely accepted that dietary lipid is an important contributor [4]. Upon ingestion of a meal, dietary lipids (predominantly triglycerides (TGs), phospholipids, and cholesterol) are hydrolyzed in the intestinal lumen and their products (free fatty acids, monoacylglycerols and free cholesterol) taken up by the enterocytes. Enterocytes re-synthesize lipids in the endoplasmic reticulum (ER) and package them for secretion as chylomicrons (CMs) [5, 6]. The hepatocytes are the site where lipids are packaged as the very low-density lipoproteins (VLDLs) and released into the circulation [7]. The CMs and VLDLs are lipoprotein particles whose assembly in the enterocytes and hepatocytes, respectively, require two major players: (a) apolipoprotein B (apoB), the structural component of the lipoproteins, and (b) microsomal triglyceride transfer protein (Mtp), an ER-resident protein that is thought to transfer lipids to apoB while the apoB transcript is being translated, thus allowing apoB to fold correctly and assemble a primordial lipoprotein particle [8–11]. Upon their maturation and secretion from the enterocytes and hepatocytes, the CMs and VLDLs serve to transport lipids to the energy-requiring tissues (muscles) or to the energy-storing adipose tissue, where the TGs in these apoB-containing lipoproteins (apoB-lipoproteins) are cleaved and the fatty acids taken up by the target tissues [12]. Mice that are deficient in *mtp*, systemically or specifically in the liver or intestine, exhibit reduction in plasma triglyceride and cholesterol levels (Chang, Liao et al., 1999, Iqbal, Parks et al., 2013, Raabe, Flynn et al., 1998, Raabe, Veniant et al., 1999), supporting the idea that dysregulation of the mechanisms that control lipid uptake and distribution could significantly perturb normal systemic lipid homeostasis.

*Drosophila* is a well-established and genetically tractable model that shares many of the same metabolic and energy-sensing pathways with vertebrates. Several models of HFD-induced obesity have been developed in *Drosophila* which recapitulated the salient features of human obesity and diabetes [13]. In *Drosophila*, Lipophorins (Lpp) are the major apoB-lipoproteins and are synthesized in the fat body, the principal fat depot and the functional equivalent of mammalian liver and adipose tissue [14]. Like the CMs and VLDLs, Lpp are also essential elements in the circulatory transport of neutral lipids and their generation in the fat body also requires Mtp and the *Drosophila* apoB homologue, apolipophorin (apoLpp) [15]. Upon their release from the fat body, Lpp can be recruited to the intestine, where they promote the absorption of dietary lipids and mediate the transport of intestinal-derived lipids to other organs for storage or energy production [15]. Flies in which Lpp are inhibited in the fat body have severely reduced levels of lipids in their circulation [15], highlighting the important role of the apoB-lipoproteins derived from the fat body in the maintenance of whole-body lipid metabolism.

The heart has been recognized in recent years to play an important role in the regulation of systemic lipid metabolism [16–19]. For instance, studies in mice and *Drosophila* have revealed that MED13, a subunit of the Mediator transcriptional complex, acts in the heart to control whole-body lipid metabolism [17–19]. Despite increasing evidence supporting the heart as a regulator of global lipid homeostasis, the underlying mechanisms remain poorly understood. The genes for Mtp and apoB are expressed in the mouse cardiomyocytes [20], where they are likely involved in the biosynthesis and secretion of apoB-lipoproteins from the heart [21, 22]. However, it is not clear whether apoB-lipoproteins derived from the heart could serve to influence systemic lipid homeostasis.

In this study using the *Drosophila* model, we reveal that the cardiomyocytes are a critical source of apoB-lipoproteins that in turn serve an equally significant role as their fat body-derived counterparts in the maintenance of systemic lipid homeostasis on normal food diet (NFD). Importantly, on high fat diet (HFD), the cardiomyocyte-derived apoB-lipoproteins are the predominant determinants of whole-body lipid metabolism. Our results further show that HFD differentially alters relative Mtp and apoLpp gene expression in the fat body and cardiomyocytes, culminating in their higher levels in the cardiomyocytes than in the fat body and possibly underpinning the differential contribution of the cardiomyocyte- versus fat body-derived apoB-lipoproteins on systemic lipid metabolic regulation under HFD conditions.

## Results and Discussion

### Genetic screening identifies Mtp as a key modulator of lipid metabolism in response to a high-fat diet

To identify genes involved in the regulation of lipid metabolism, we performed a haplo-insufficiency screen for genes that could alter the pathogenesis of HFD-induced obesity in *Drosophila* (S1A Fig). We first developed a feeding regimen in which control *w*^*1118*^ female and male flies were reared on a NFD or a HFD (~48% of the calories derived from Crisco hydrogenated vegetable oils) (S1B and S1B′ Fig) until adult eclosion. Measurement of whole-body triglyceride (TG) levels in the newly eclosed adults revealed a significant increase (~1.5 fold) in TG levels in HFD-reared compared to the NFD-reared progeny (S1C Fig). Consistent with that, Nile Red staining of the fat body revealed that control *w*^*1118*^ HFD-fed larvae had increased neutral lipid accumulation (S1D Fig, arrows) in the adipocytes (S1D Fig, outlines) compared to NFD-fed larvae. Collectively, these data provide evidence that our HFD feeding regimen generates a *Drosophila* model of diet-induced obesity.

**Fig 1:**
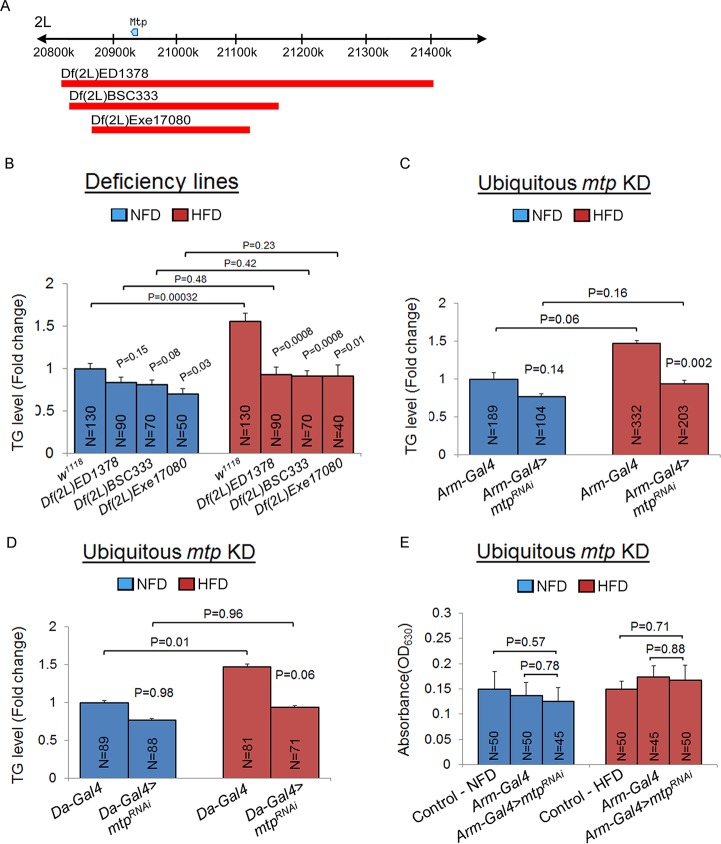
Genetic screening identifies Mtp as a gene for determining systemic lipid metabolism. (A) Schematic of the results of the genetic screen showing the location of the *mtp* gene (blue symbol) on chromosome 2L. Red bars indicate deletions in the three deficiency lines investigated here: *Df(2L)ED1378*, *Df(2L)BSC333*, and *Df(2L)Exel7080*. (B) Whole-body TG levels of newly eclosed control (*w*^*1118*^) flies and deficiency lines on NFD or HFD. For each genotype, a 1:1 ratio of males:females was analyzed. *P*-values are from Student’s *t*-test and are between control *w*^*1118*^ and deficiency lines within NFD or HFD, or between NFD and HFD for the same genotype. (C) Whole-body TG levels of newly eclosed control flies (*Arm-Gal4*), and flies with whole-body KD of *mtp* (*Arm-Gal4>mtp*^*RNAi*^) on NFD and HFD. For each genotype, a 1:1 ratio of males:females was analyzed. *P*-values are from Student’s *t*-test and are between *Gal4* control and *Gal4*-mediated RNAi lines within NFD or HFD, or between NFD and HFD for the same genotype. (D) Whole-body TG levels of newly eclosed control flies (*Da-Gal4*), and flies with whole-body KD of *mtp* (*Da-Gal4>mtp*^*RNAi*^) on NFD and HFD. For each genotype, a 1:1 ratio of males:females was analyzed. *P*-values are from Student’s *t*-test and are between *Gal4* control and *Gal4*-mediated RNAi lines within NFD or HFD, or between NFD and HFD for the same genotype. In (B), (C) and (D), TG levels (μg/μl) were normalized to total protein (μg/μl). Results are expressed as the fold change in whole fly normalized TG compared with that of the wild-type *w*^*1118*^ or *Gal4* control flies on NFD (set to 1.0). Results are the mean ± SEM of the indicated number of flies (N) analyzed over at least 5 independent experiments. (E) Colorimetric assay of food intake in control *w*^*1118*^ third instar larvae and third instar larvae with *Gal4* driver only (*Arm-Gal4*) or with whole body knockdown of *mtp* using *Arm-Gal4* (*Arm-Gal4>mtp*^*RNAi*^). Results are the mean ± SEM of the indicated number of larvae (N) analyzed over 3 independent experiments. *P*-values are from Student’s *t*-test.

Using the same feeding regimen to screen a collection of deficiency lines, we identified three overlapping deficiency lines, *Df(2L)ED1378*, *Df(2L)BSC333*, and *Df(2L)Exel7080* within chromosome 2L that displayed significant decreases in TG levels compared to control *w*^*1118*^ flies on HFD (Fig 1A and 1B). Moreover, the TG levels in these deficiency lines under NFD conditions are comparable to those under HFD conditions (Fig 1B). Analysis of the deficiency lines revealed that *mtp* is included in the gene deletions in all three deficiency lines (Fig 1A). As a role of Mtp in the control of lipid levels has previously been shown in *Drosophila* [15], we tested whether inhibition of Mtp could cause a resistance in the HFD-induced gain in TG levels, by performing RNA interference (RNAi)-mediated knockdown (KD) of *mtp* using the ubiquitous *Armadillo-Gal4* (*Arm-Gal4*) and *Daughterless-Gal4* (*Da-Gal4*) drivers. We found that newly-eclosed whole flies that ubiquitously expressed *mtp* RNAi (*Arm-Gal4>mtp*^*RNAi*^ or *Da-Gal4>mtp*^*RNAi*^) displayed significantly (Fig 1C) or near-significantly (Fig 1D) lower systemic TG levels than *Arm-Gal4* or *Da-Gal4* control flies on HFD. Moreover, the *Arm-Gal4>mtp*^*RNAi*^ or *Da-Gal4>mtp*^*RNAi*^ flies did not show any significant elevation in overall TG levels on HFD compared to NFD (Fig 1C and 1D). Such suppression in the gain in systemic TG content associated with Mtp inhibition was not due to changes in feeding behavior, as the *Arm-Gal4>mtp*^*RNAi*^ larvae had similar food consumption as the control *w*^*1118*^ or control *Arm-Gal4* larvae on NFD or HFD (Fig 1E). Taken together, these results support a role for Mtp in the control of systemic lipid metabolism.

### Inhibition of Mtp in fat body and cardiomyocytes affect normal systemic triglyceride levels

**Fig 2:**
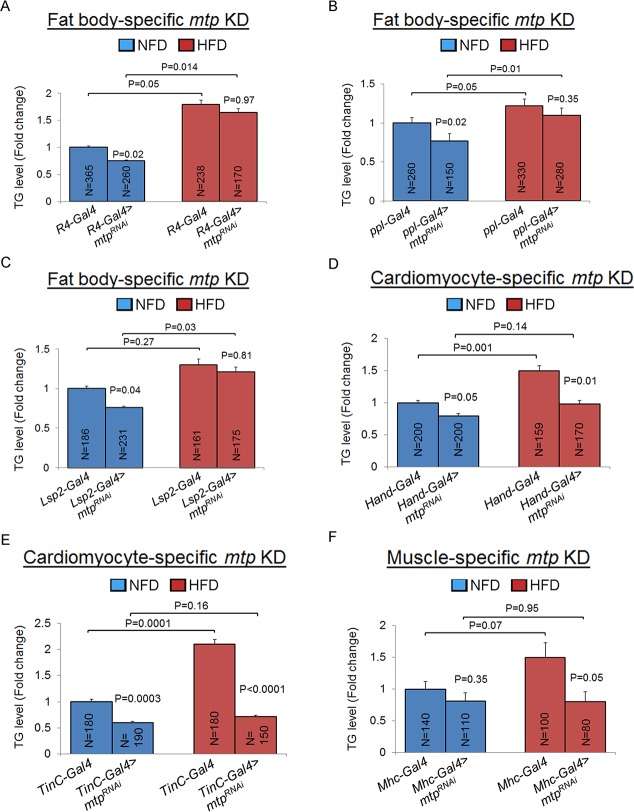
Fat body and cardiomyocyte Mtp regulate systemic lipid metabolism differently on normal food diet and high fat diet. (A–C) Whole-body TG levels of newly-eclosed flies with *Gal4* drivers only (controls) or with fat body-specific knockdown of *mtp* using *R4-Gal4* (A), *ppl-Gal4* (B), or *Lsp2-Gal4* (C) on NFD (blue) or HFD (red). (D-E) Whole-body TG levels of newly-eclosed flies with *Gal4* drivers only (controls) or with cardiomyocyte-specific knockdown of *mtp* using *Hand-Gal4* (D), or *TinC-Gal4* (E) on NFD (blue) or HFD (red). (F) Whole-body TG levels of newly-eclosed flies with *Gal4* drivers only (controls) or with muscle-specific knockdown of *mtp* using *Mhc-Gal4* on NFD (blue) or HFD (red). In all cases, TG levels (μg/μl) were normalized to total protein (μg/μl). Results are expressed as the fold change in whole fly normalized TG compared with that of the *Gal4* control flies on NFD (set to 1.0). Results are the mean ± SEM of the indicated number of flies (N) analyzed over at least 5 independent experiments. *P*-values are from Student’s *t*-test and are between *Gal4* control and *Gal4*-mediated RNAi lines within NFD or HFD, or between NFD and HFD for the same genotype.

Mtp was reported to function in the fat body to promote the generation of apoB-lipoproteins from the fat body [15]. We therefore determined whether the fat body could be the major or sole tissue that Mtp acts in to regulate whole-body lipid metabolism, by probing the tissue-specific contribution of Mtp via targeted RNAi-mediated *mtp* KD in different tissues followed by analyses of whole-body TG levels on NFD. S1 Table provides a summary of the different Gal4 drivers used to induce mtp KD and their effects on developmental timing and viability. We found that KD of *mtp* specifically in the fat body using three different fat body-specific *Gal4* drivers (S2A and S2B Fig) led to a significant reduction in whole-body TG levels compared to their respective controls under NFD condition (Fig 2A–2C; S3A Fig). However, strikingly, when we KD *mtp* specifically in the cardiomyocytes using the myocardial-specific *Hand-* and *TinC-Gal4* drivers, we also found a significant lowering of whole-body TG levels in the newly-eclosed adult flies relative to their respective controls under NFD condition (Fig 2D and 2E). On the other hand, targeted KD of *mtp* in the nonmyocytic pericardial cells of the *Drosophila* heart [23], using the *Sticks-n-stones* (*Sns)*-*Gal4* driver [24], did not significantly affect systemic TG levels in the newly-eclosed adult KD flies relative to control flies on NFD (S4A Fig). In addition, we found that KD of *mtp* specifically in the central nervous system or intestine had no effect on whole-body TG levels compared to the respective controls on NFD in the newly-eclosed adult flies (S4B and S4C Fig). Taken together, these findings reveal a previously-unrecognized role of Mtp in the cardiomyocytes in regulating whole-body lipid metabolism under NFD conditions.

### Cardiomyocyte Mtp is the main determinant of whole-body lipid metabolism on high-fat diet

We next asked whether Mtp in the fat body and/or cardiomyocytes could serve similar roles in the regulation of systemic lipid metabolism under HFD conditions. To test that, we performed targeted KD of *mtp* in the fat body and assessed whole-body TG levels on HFD. We postulated that if Mtp serves a similar function in the fat body on whole-body lipid metabolic regulation on HFD as on NFD, then total TG levels would be lower in the *mtp* KD flies relative to control flies under HFD condition. Unexpectedly, the KD of *mtp* in fat body led to an increase in total TG content comparatively to control flies in response to high dietary fat intake (Fig 2A–2C; S3A Fig), suggesting that while Mtp in the fat body critically regulates systemic lipid metabolism on NFD, it does not influence lipid metabolic responses to HFD. In contrast, silencing of *mtp* specifically in the cardiomyocytes led to a significant decrease in whole-body TG levels relative to the control on HFD, as seen on NFD (Fig 2D and 2E). In whole larvae, whereas targeted KD of *mtp* in the cardiomyocytes using *Hand-Gal4* did not elicit any significant change in systemic TG level compared to the control on HFD (S3B Fig), there was a trend of whole-body TG content decrease in larvae in which mtp silencing in the cardiomyocyte was directed using *TinC-Gal4* (S3C Fig). We also targeted *mtp* KD specifically in the cardiomyocytes and skeletal muscles using the muscle-specific *Mhc-Gal4* driver and again found a significant down-regulation of systemic TG levels in the *Mhc-Gal4>mtp*^*RNAi*^ flies compared to the control on HFD (Fig 2F). On the other hand, inhibition of Mtp in the pericardial cells, central nervous system or intestine did not significantly alter whole-body TG levels relative to the controls on HFD (S4A–S4C Fig). In sum, these results indicate that Mtp in the cardiomyocytes serves a predominant role in the regulation of systemic lipid metabolism under HFD condition.

One possibility for the HFD-dominant effect of the cardiomyocytes on systemic lipid metabolism could be due to HFD-induced cardiac dysfunction, which has been previously reported in adult flies fed on a coconut oil HFD [25]. For instance, HFD-induced cardiac dysfunction could perturb hemolymph flow and elicit more extensive systemic effects on lipid metabolism. To investigate this possibility, we examined the cardiac contractility of wandering third-instar larvae that were fed, in our case, on NFD or HFD for about 4 days (after egg hatching) (S1A Fig). Although such short-term feeding on HFD only during the larval stage was sufficient to induce significant lipid accumulation in the larval fat body (S1D Fig), it caused only mild levels of lipid deposition in the heart compared to NFD-fed larvae (S5A Fig). Moreover, there was no significant difference between NFD- and HFD-fed larvae in terms of heart period (S5B and S5C Fig), heartbeat regularity (S4D Fig) and fractional shortening (S5E Fig), cardiac contractility parameters that are likely important regulators of hemolymph flow. There were also no significant differences in heart tube dimensions between the HFD- and NFD-fed larvae during cardiac relaxation (S5F Fig) and contraction (S5G Fig). We conclude that the cardiomyocytes modulate systemic lipid metabolism in a manner that is independent of cardiac performance under HFD conditions.

It was previously shown that Mtp could play a role during *Drosophila* trachea development [26]. This prompted us to examine whether Mtp could also regulate proper heart development and function which in turn modulates systemic lipid metabolism. To test that, we targeted the KD of *mtp* in cardiomyocytes using *Hand-Gal4* and performed heart functional analyses on the newly eclosed adult flies, a stage when lipid metabolic alterations were already apparent in flies with *mtp* KD in the heart (Fig 2D–2F). We found that there were no significant differences between the control and *Hand-Gal4>mtp*^*RNAi*^ flies in their heart period (S6A Fig), heart rhythm (S6B Fig), heart tube dimensions during cardiac relaxation (S6C Fig) and contraction (S6D Fig), or fractional shortening (S6E Fig) on NFD or HFD. As the inhibition of Mtp in the cardiomyocytes had no obvious effects on any major aspects of heart function on under either diet condition, our results suggest that Mtp in the cardiomyocytes regulates systemic lipid metabolism in a manner that is independent of any potential influence of Mtp on cardiac performance maintenance.

### Lipophorins mediate the effects of Mtp in fat body and cardiomyocytes on systemic lipid metabolism

**Fig 3:**
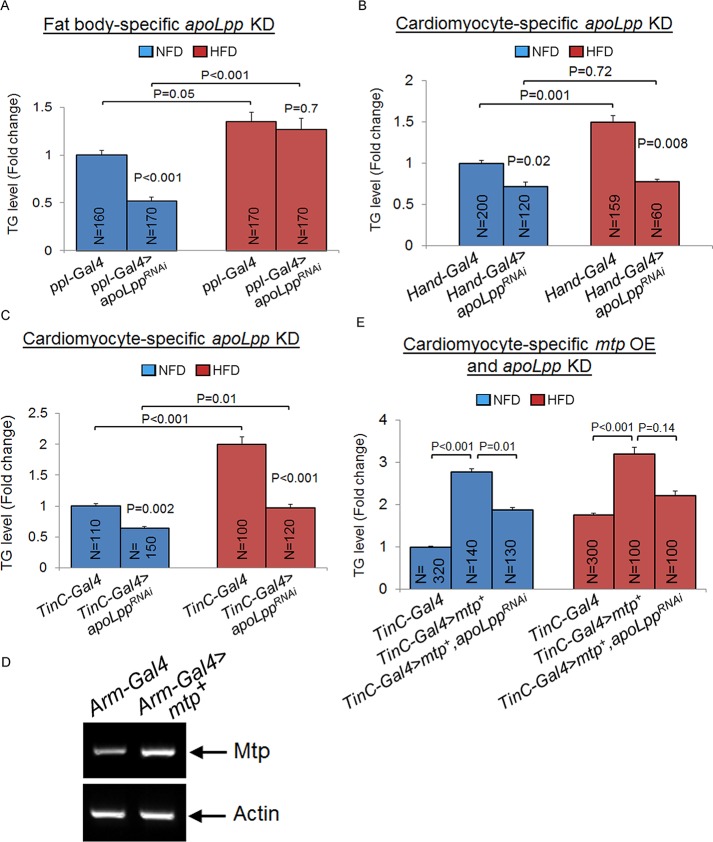
Fat body and cardiomyocyte apoLpp regulate systemic lipid metabolism differently on normal food diet and high fat diet. (A) Whole-body TG levels of newly-eclosed flies with *Gal4* driver only (control) or with fat body-specific knockdown of *apoLpp* using *ppl-Gal4* (A) on NFD (blue) or HFD (red). (B, C) Whole-body TG levels of newly-eclosed flies with *Gal4* drivers only (controls) or with cardiomyocyte-specific knockdown of *apoLpp* using *Hand-Gal4* (B), or *TinC-Gal4* (C) on NFD (blue) or HFD (red). (D) RT-PCR analysis of *mtp* mRNA levels in whole flies with *Arm-Gal4* driver only (control) or with *Arm-Gal4*-induced overexpression of full-length *mtp* (*mtp*^*+*^). Actin serves as an internal control. (E) Whole-body TG levels of newly-eclosed flies with *Gal4* driver only (control), with cardiomyocyte-specific overexpression of *mtp*^*+*^ using *TinC-Gal4*, or with cardiomyocyte-specific overexpression of *mtp*^*+*^ and *apoLppRNAi* using *TinC-Gal4* on NFD (blue) or HFD (red). In A-C and E, TG levels (μg/μl) were normalized to total protein (μg/μl). Results are expressed as the fold change in whole fly normalized TG compared with that of the *Gal4* control flies on NFD (set to 1.0). Results are the mean ± SEM of the indicated number of flies (N) analyzed over at least 5 independent experiments. *P*-values are from Student’s *t*-test and are between *Gal4* control and *Gal4*-mediated RNAi lines within NFD or HFD, or between NFD and HFD for the same genotype.

Mtp is an essential factor in the assembly and secretion of the major apoB-lipoproteins termed lipophorins or Lpp in flies [15, 27]. If Mtp exerts its effect on systemic lipid metabolism via Lpp, we would expect that inhibition of apoLpp (protein scaffold of Lpp and another essential factor in Lpp biosynthesis) would recapitulate the systemic lipid metabolic effects associated with the silencing of *mtp*. Indeed, as shown in Fig 3A, the fat body-targeted KD of *apoLpp* led to a significant lowering of whole-body TG levels on NFD but not on HFD relative to the control, metabolic effects that recapitulated those seen in the fat body-specific *mtp* KD flies under NFD and HFD conditions (Fig 2A–2C). Next, we performed the KD of *apoLpp* only in the cardiomyocytes using *Hand-Gal4* (S2C Fig) and *TinC-Gal4* drivers and found that that resulted in significantly lower levels of systemic TG levels compared to the controls on NFD or HFD (Fig 3B and 3C; S3B and S3C Fig), metabolic effects that are reminiscent of those associated with *mtp* silencing in the cardiomyocytes under the different diet conditions (Fig 2D–2F). We further investigated the epistatic relationship between Mtp and apoLpp on systemic lipid metabolic control in the cardiomyocytes. We first generated a *UAS-mtp*^*+*^ fly line which when crossed to flies bearing the *Arm-Gal4* driver resulted in an approximate two-fold increase in wild-type *mtp* expression throughout the body (Fig 3D). We then crossed *UAS-mtp*^*+*^ flies to flies harboring the cardiomyocyte-specific *TinC*-*Gal4* driver, and found that the progenies (*TinC-Gal4>mtp*^*+*^) displayed significant elevation in their TG levels compared to the control on NFD or HFD (Fig 3E). Notably, these effects were alleviated by the concurrent inhibition of apoLpp in the cardiomyocytes (*TinC-Gal4>mtp*^*+*^,*apoLpp*^*RNAi*^) (Fig 3E). We conclude that Mtp- and apoLpp-dependent generation of Lpp in the fat body and cardiomyocytes serves to critically regulate systemic lipid metabolism under NFD and HFD conditions.

### Cardiomyocyte-derived lipophorins control dietary lipid uptake from the enterocytes and fat mobilization in the fat body

**Fig 4:**
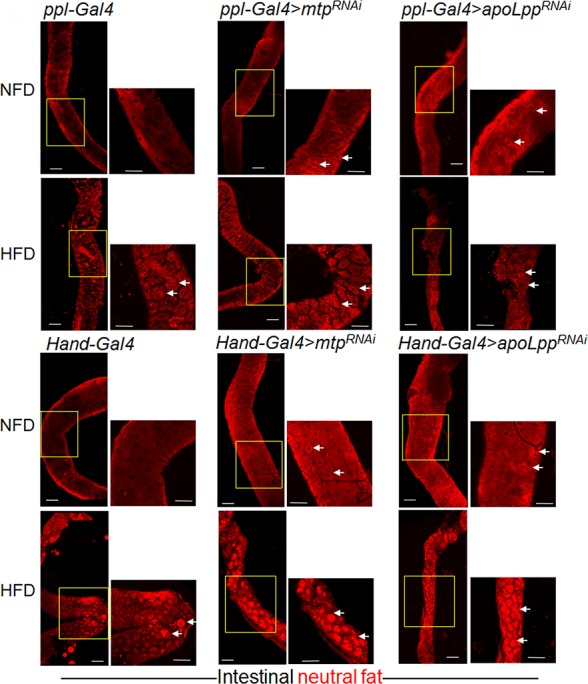
Inhibition of Mtp or apoLpp in fat body or cardiomyocytes promotes intestinal lipid accumulation on normal food diet and high fat diet. (A-C’) Representative confocal images of Nile Red-stained lipid droplets in the unfixed intestines of third instar larvae on NFD. (A, A’), control larvae (*ppl-Gal4*); (B, B’), larvae with fat body-specific KD of *mtp* (*ppl-Gal4>mtp*^*RNAi*^); and (C, C’), larvae with fat body-specific KD of *apoLpp* (*ppl-Gal4>apoLpp*^*RNAi*^). (A, B, C) Lower magnification (10X) images with scale bars representing 100 μm. (A’, B’, C’) Insets represent the magnified (20X) portion of the intestines (yellow boxes) with scale bar representing 70 μm. Arrows in insets indicate lipid droplets within the enterocytes. (D-F’) Representative confocal images of Nile Red-stained lipid droplets in the unfixed intestines of third instar larvae on HFD. (D, D’), control larvae (*ppl-Gal4*); (E, E’), larvae with fat body-specific KD of *mtp* (*ppl-Gal4>mtp*^*RNAi*^); and (F, F’), larvae with fat body-specific KD of *apoLpp* (*ppl-Gal4>apoLpp*^*RNAi*^). (D, E, F) Lower magnification (10X) images with scale bars representing 100 μm. (D’, E’, F’) Insets represent the magnified (20X) portion of the intestines (yellow boxes) with scale bar representing 70 μm. Arrows in insets indicate lipid droplets within the enterocytes. (G-I’) Representative confocal images of Nile Red-stained lipid droplets in the unfixed intestines of third instar larvae on NFD. (G, G’), control larvae (*Hand-Gal4*); (H, H’), larvae with cardiomyocyte-specific KD of *mtp* (*Hand-Gal4>mtp*^*RNAi*^); and (I, I’), larvae with cardiomyocyte-specific KD of *apoLpp* (*Hand-Gal4>apoLpp*^*RNAi*^). (G, H, I) Lower magnification (10X) images with scale bars representing 100 μm. (G’, H’, I’) Insets represent the magnified (20X) portion of the intestines (yellow boxes) with scale bar representing 70 μm. Arrows in insets indicate lipid droplets within the enterocytes. (J-L’) Representative confocal images of Nile Red-stained lipid droplets in the unfixed intestines of third instar larvae on HFD. (J, J’), control larvae (*Hand-Gal4*); (K, K’), larvae with cardiomyocyte-specific KD of *mtp* (*Hand-Gal4>mtp*^*RNAi*^); and (L, L’), larvae with cardiomyocyte-specific KD of *apoLpp* (*Hand-Gal4>apoLpp*^*RNAi*^). (J, K, L) Lower magnification (10X) images with scale bars representing 100 μm. (J’, K’, L’) Insets represent the magnified (20X) portion of the intestines (yellow boxes) with scale bar representing 70 μm. Arrows in insets indicate lipid droplets within the enterocytes.

Next, we asked how the cardiomyocytes could modulate lipid metabolism in a systemic manner. A previous study found that inhibition of Mtp or apoLpp in the fat body caused lipid accumulation in the intestine on NFD, suggesting that Lpp could be recruited to the intestine from the fat body for the uptake of dietary lipids into the circulation [15]. We found that KD of *mtp* or *apoLpp* specifically in the fat body, using the *ppl-Gal4* driver, increased the number of neutral lipid droplets in the larval intestines compared to control intestines on NFD (Fig 4A–4C’; see arrows in Fig 4B’ and 4C’), consistent with the previous study [15]. However, under HFD condition, there was no significant difference in the levels of neutral lipids in the larval intestine between the *mtp* KD and control flies (Fig 4D and 4E’), or between the apoLpp KD and control flies (Fig 4D and 4D’ and 4F and 4F’), corroborating the notion that Lpp from the fat body do not impact systemic lipid metabolism under HFD condition (Fig 2A and 2B and Fig 3A).

We next sought to determine the mechanism by which cardiomyocyte-derived Lpp regulate systemic lipid metabolism. We found that under NFD conditions, targeted inhibition of Mtp or apoLpp in the cardiomyocytes using the *Hand-Gal4* driver (Fig 4G–4I’) also led to an increase in larval intestinal lipid accumulation compared to control intestines (see arrows in Fig 4H’ and 4I’), thereby supporting a similarly important role of the cardiomyocyte- and fat body-derived Lpp in the regulation of systemic lipid metabolism via mediating the uptake of dietary lipids from the intestine. On HFD however, whereas the inhibition of Mtp or apoLpp specifically in the fat body did not significantly affect intestinal lipid levels compared to control intestines (Fig 4D–4F’), the cardiomyocyte-specific *mtp* or *apoLpp* KD larvae still exhibited an increase in intestinal lipid content relative to control intestines (Fig 4J–4L’; see arrows in Fig 4K’ and 4L’). Such accumulation of lipids in the intestines in the cardiomyocyte-specific *mtp* or *apoLpp* KD larvae under HFD conditions was associated with a reduction in intestinal Lpp abundance, as indicated by their decreased intestinal apoLpp staining relative to control intestines (S7A–S7C Fig). These results thereby provide evidence that on HFD, the cardiomyocyte-derived Lpp serve as major determinants of systemic lipid metabolism via their recruitment to the intestine to promote the circulatory absorption of dietary lipids.

### High-fat diet alters relative Mtp levels in the cardiomyocytes and fat body

**Fig 5:**
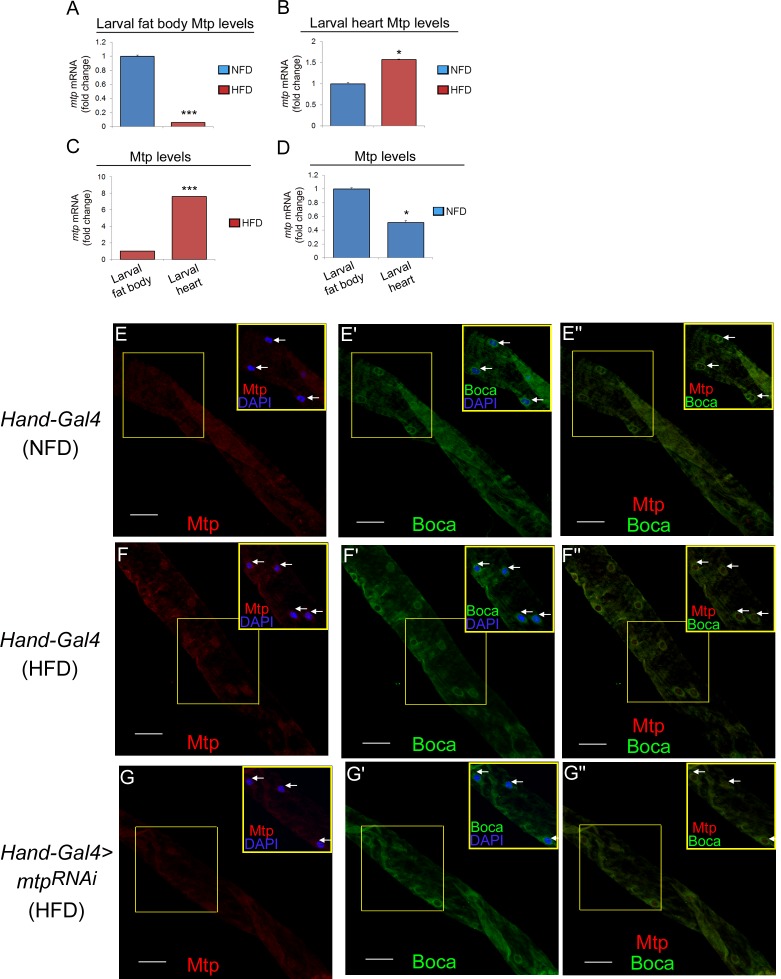
High fat diet alters relative Mtp expression levels in the cardiomyocytes and fat body. (A, B) Relative mRNA levels of Mtp in larval fat body (A) and larval hearts (B) on NFD and HFD. Results are expressed as the fold difference compared with the NFD condition. Values were normalized with *gapdh*. ***p < 0.001, by two-tailed paired *t*-test analyzed over 3 independent experiments. (C) Relative mRNA levels of Mtp in in larval heart relative to larval fat body on HFD. Results are expressed as the fold difference. Values were normalized with gapdh. *p < 0.05, ***p < 0.001, by two-tailed paired *t*-test analyzed over 3 independent experiments. (D) Relative mRNA levels of Mtp in larval heart relative to larval fat body on NFD. Results are expressed as the fold difference. Values were normalized with gapdh. *p < 0.05, ***p < 0.001, by two-tailed paired *t*-test analyzed over 3 independent experiments. (E-E”) Representative confocal images of Mtp (E), Boca (E’), and Mtp and Boca (E”) stainings of the hearts in the *Hand-Gal4* control third instar larvae on NFD. Scale bars represent 40 μm. Insets in E-E” represent the same portion of the heart tube (yellow boxes). Arrows in inset E mark Mtp and DAPI nuclear staining in three cardiomyocytes whereas arrows in inset E’ and inset E” mark Boca and DAPI staining and Mtp and Boca staining in the same three cardiomyocytes, respectively. (F-F”) Representative confocal images of Mtp (F), Boca (F’), and Mtp and Boca (F”) stainings of the hearts in the *Hand-Gal4* control third instar larvae on HFD. Scale bars represent 40 μm. Insets in F-F” represent the same portion of the heart tube (yellow boxes). Arrows in inset F mark Mtp and DAPI nuclear staining in four cardiomyocytes whereas arrows in inset F’ and inset F” mark Boca and DAPI staining and Mtp and Boca staining in the same four cardiomyocytes, respectively. (G-G”) Representative confocal images of Mtp (G), Boca (G’), and Mtp and Boca (G”) stainings of the hearts in the *Hand-Gal4*−mediated *mtp* KD third instar larvae on HFD. Scale bars represent 40 μm. Insets in G-G” represent the same portion of the heart tube (yellow boxes). Arrows in inset C mark Mtp and DAPI nuclear staining in two cardiomyocytes whereas arrows in inset G’ and inset G” mark Boca and DAPI staining and Mtp and Boca staining in the same two cardiomyocytes, respectively.

To understand the HFD-associated predominant role of cardiomyocyte-derived Lpp mechanistically, we investigated the expression levels of Mtp and apoLpp under NFD and HFD conditions. We hypothesized that Mtp and/or apoLpp gene expression differentially changes in the cardiomyocytes and fat body such that their expression levels are significantly higher in the cardiomyocytes than in fat body in response to abundant dietary fat intake. Therefore, the KD of *mtp* or *apoLpp* in the cardiomyocytes and not the fat body will have an obvious effect on systemic lipid metabolism under HFD condition. To test this idea, we first analyzed Mtp mRNA expression in the cardiomyocytes and fat body on NFD or HFD using quantitative RT-PCR. First of all, we found that upon initiation of HFD feeding after egg hatching (S1A Fig), changes in the relative expression of Mtp could be detected as early as in the wandering third instar larval stage, about 4 days later (Fig 5). Strikingly, relative to NFD, HFD robustly down-regulated Mtp transcript level by 16.7-fold in the larval fat body (Fig 5A) but strongly up-regulated Mtp transcript level by 1.57-fold in the larval cardiomyocytes (Fig 5B). Inter-tissue comparison further revealed that there was a significant 7.6-fold higher Mtp transcript level in the cardiomyocytes than in fat body under HFD condition (Fig 5C), although normal transcript level of Mtp was lower in the cardiomyocytes relative to the fat body (Fig 5D). We further determined whether the changes in the Mtp gene expression in the fat body versus cardiomyocytes under NFD and HFD conditions would also occur at the protein level. Indeed, Mtp protein content in the larval fat body decreased under HFD condition relative to NFD condition (S8A Fig), consistent with the change at mRNA level (Fig 5A). We next compared NFD and HFD Mtp levels in the larval cardiomyocytes by direct visualization of these proteins using immunofluorescence. We observed that Mtp was weakly expressed in the cardiomyocytes on NFD (Fig 5E, arrows in inset) but its expression was increased in response to HFD (Fig 5F, arrows in inset), in line with the gene expression changes (Fig 5B). Cardiac-specific knockdown of *mtp* reduced the immunofluorescence of Mtp in the cardiomyocytes on HFD (Fig 5G, arrows in inset), indicative of the staining specificity of the Mtp antibody. We further found that on HFD, Mtp partially localized with Boca, an endoplasmic reticulum (ER) marker [28], in the cardiomyocytes (Fig 5F–5F”, compare arrows in insets 5F and 5F’), thus establishing that Mtp in the *Drosophila* cardiomyocytes resides, in part, in the ER.

### High-fat diet alters relative apoLpp levels in the cardiomyocytes and fat body

**Fig 6:**
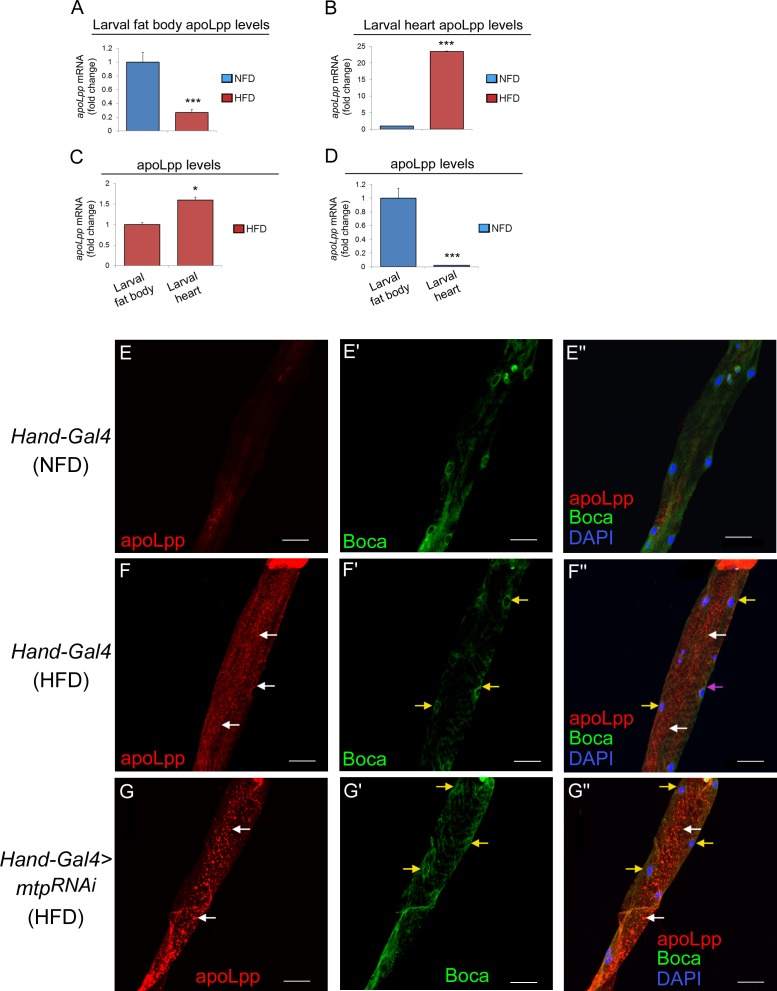
High fat diet alters relative apoLpp expression levels in the cardiomyocytes and fat body. (A, B) Relative mRNA levels of apoLpp in larval fat body (A) and larval hearts (B) on NFD and HFD. Results are expressed as the fold difference compared with the NFD condition. Values were normalized with *gapdh*. ***p < 0.001, by two-tailed paired *t*-test analyzed over 3 independent experiments. (C) Relative mRNA levels of apoLpp in in larval heart relative to larval fat body on HFD. Results are expressed as the fold difference. Values were normalized with gapdh. *p < 0.05, ***p < 0.001, by two-tailed paired *t*-test analyzed over 3 independent experiments. (D) Relative mRNA levels of apoLpp in larval heart relative to larval fat body on NFD. Results are expressed as the fold difference. Values were normalized with gapdh. *p < 0.05, ***p < 0.001, by two-tailed paired *t*-test analyzed over 3 independent experiments. (E-E”) Representative confocal images of apoLpp (E), Boca (E’), and apoLpp, Boca and DAPI (E”) in cardiomyocytes of the *Hand-Gal4* control third instar larvae on NFD. Scale bars represent 40 μm. Boca marks the endoplasmic reticulum and DAPI marks the nucleus in each cardiomyocyte. (F-F”) Representative confocal images of apoLpp (F), Boca (F’), and apoLpp, Boca and DAPI (F”) in cardiomyocytes of the *Hand-Gal4* control third instar larvae on HFD. White arrows in F and F” indicate the apoLpp puncta which reflect the presence of Lpp. Yellow arrows in F’ and F” indicate the endoplasmic reticulum marker Boca, and DAPI marks the nuclei in the cardiomyocytes. The magenta arrow in F” indicates the co-localization of apoLpp and Boca. Scale bars represent 40 μm. (G-G”) Representative confocal images of apoLpp (G), Boca (G’), and apoLpp, Boca and DAPI (G”) in cardiomyocytes of the *Hand-Gal4*-mediated *mtp* KD third instar larvae on HFD. White arrows in G and G” indicate the strong punctate staining of apoLpp which reflects the accumulation of Lpp. Yellow arrows in G’ and G” indicate the endoplasmic reticulum marker Boca, and DAPI marks the nuclei in the cardiomyocytes. Scale bars represent 40 μm.

Next, we examined whether HFD also differentially alters apoLpp expression in the cardiomyocytes versus the fat body, as seen for Mtp (Fig 5). Our results showed that apoLpp transcript level was decreased on HFD compared to NFD in the larval fat body, by about 3.75 fold (Fig 6A). In contrast, relative to NFD, HFD dramatically increased apoLpp mRNA level by 23.5 fold in the cardiomyocytes (Fig 6B), to a much greater extent than Mtp transcript level activation in the same tissue (Fig 5B). Inter-tissue comparison revealed a significant 1.6-fold increase in apoLpp transcript level in the cardiomyocytes compared to the fat body under HFD condition (Fig 6C), even though NFD apoLpp transcript level was lower in the cardiomyocytes than in fat body (Fig 6D). At the protein level, we found that apoLpp concentration in the fat body was reduced in response to high dietary fat feeding (S8B Fig), in agreement with the alteration in mRNA level (Fig 6A). Our examination of apoLpp directly in the cardiomyocytes also revealed a higher expression level of apoLpp on HFD than on NFD, as shown by the stronger punctate staining of apoLpp on HFD (Fig 6F, white arrows) relative to NFD (Fig 6E). Cardiac-specific knockdown of apoLpp abolished the punctate staining of apoLpp in the cardiomyocytes on HFD (S2C Fig), indicative of the staining specificity of the apoLpp antibody. Unlike Mtp, minimal co-localization of apoLpp and Boca was detected in the cardiomyocytes (Fig 6F”, arrows), suggesting that apoLpp was dynamically distributed throughout the cell likely due to its association with lipoproteins. In all, our Mtp and apoLpp expression data support our above hypothesis that HFD exerts an opposing effect on relative Mtp and apoLpp levels in the cardiomyocytes and fat body that could contribute to the predominant role of cardiomyocyte-derived Lpp in determining systemic lipid metabolic responses to HFD.

### Cardiomyocyte-derived Lpp constitute a significant portion of total Lpp pool in the circulation under high-fat diet condition

One possible outcome of the above-seen HFD-induced differential expression of Mtp and apoLpp expression in the fat body versus the cardiomyocytes could be a decreased and increased production of Lpp from the fat body and cardiomyocytes, respectively. In that case, we reasoned that inhibition of the Lpp biosynthesis genes only in the cardiomyocytes on HFD would significantly impact total Lpp abundance in the circulation, whereas disruption of those genes only in the fat body on HFD should have a minimal impact. To test this, we determined the total amount of circulating Lpp via western blot analysis of apoLpp (protein scaffold of Lpp) upon the silencing of *mtp* specifically in the fat body (using *ppl-Gal4*) or in the cardiomyocytes (using *Hand-Gal4*) in third-instar larvae on NFD and HFD. As shown in S9 Fig, inhibition of Mtp only in the fat body led to a detectable reduction in hemolymph Lpp level compared to the control on NFD (S9A Fig) but not on HFD (S9B Fig). Conversely, targeted disruption of *mtp* in the cardiomyocytes significantly attenuated the hemolymph concentration of Lpp compared to the control on HFD (S9D Fig) while eliciting a modest effect on NFD (S9C Fig). We further argue that the diminished level of hemolymph Lpp upon KD of *mtp* in the cardiomyocytes on HFD is a corollary of the reduced ability of the *mtp*-silenced cardiomyocytes to secrete Lpp, as Mtp is essential for the cellular release of Lpp [15, 27]. If so, more Lpp should be retained in the *mtp*-silenced cardiomyocytes compared to control cardiomyocytes on HFD. In agreement with this, we observed strong accumulation of apoLpp in the *mtp*-silenced cardiomyocytes relative to control cardiomyocytes in response to high dietary fat (compare Fig 6F and 6G, arrows), which correlates with the reduced hemolymph level of Lpp in the cardiomyocyte-specific *mtp* KD larvae over control larvae on HFD (S9D Fig). These results corroborate our genetic data in supporting a particularly crucial function of Mtp in the cardiomyocytes in promoting the cardiac release of Lpp into the circulation during HFD condition, demonstrating that cardiomyocyte Mtp serves as the predominant determinant of HFD-induced systemic lipid metabolic responses.

**Fig 7:**
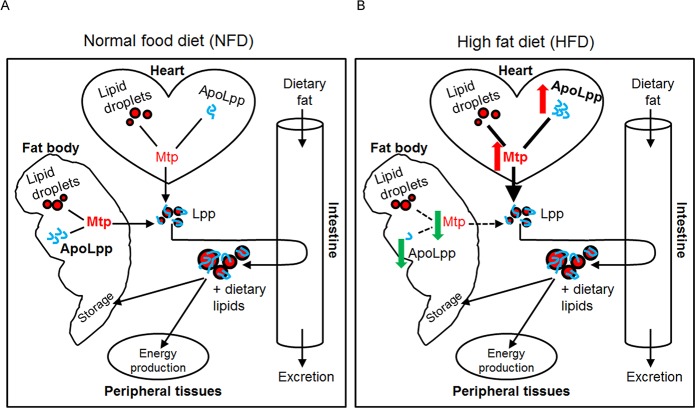
A model depicting the relative contributions of Lpp derived from the fat body and cardiomyocytes in controlling systemic lipid metabolism on NFD and HFD. (A) On NFD, Lpp derived from the cardiomyocytes play an equally important role as Lpp derived from the fat body in systemic lipid homeostasis maintenance. Higher levels of Mtp and apoLpp (blue threads) are present in the fat body than in cardiomyocytes on NFD. Both the cardiomyocyte- and fat body-derived Lpp are recruited to the intestine where they promote the uptake of dietary lipids from the enterocytes and transport the dietary lipids to peripheral tissues for energy production or for storage in the fat body. (B) HFD induces an upregulation of the relative expression of Mtp and apoLpp in the cardiomyocytes (red arrows) and downregulation of their relative expression in the fat body (green arrows), culminating in higher levels of Mtp (bold) and apoLpp (blue threads) in the cardiomyocytes than in the fat body. This could underlie the predominant role of the cardiomyocyte-derived Lpp in the determining of lipid metabolic responses to HFD (thick versus broken arrows, B).

Several factors have recently been identified to act in the heart to control systemic lipid homeostasis, such as the atrial natriuretic peptide/brain natriuretic peptide and the Mediator component Med13 [16, 17]. These findings define the heart as an important organ in regulating whole-body lipid metabolism and are beginning to unravel the mechanisms by which the heart regulates systemic lipid homeostasis. Our study now uncovers that in *Drosophila*, the cardiomyocytes are a critical source of apoB-lipoproteins that in turn serve an equally significant role as their fat body-derived counterparts in the maintenance of systemic lipid homeostasis on NFD (Fig 7A). Importantly, under HFD conditions, the cardiomyocyte-derived apoB-lipoproteins are the predominant determinants of whole-body lipid metabolism (Fig 7B). These findings provide new insight into the mechanisms that mediate the heart control of systemic lipid metabolism, and it remains to be determined whether the different cardiac-derived factors could act in concert to regulate global lipid metabolism; for instance, whether Med13 could influence Mtp or apoLpp expression in the heart under different diet conditions.

In consideration of the understanding that TG levels are very dependent on genetic background, in this study, we analyzed the TG/protein ratio between each driver *Gal4* control flies and the same *Gal4*>*UAS-RNAi* knockdown flies that are progenies derived from a single cross. We clearly observed an alteration in normalized TG levels when comparing between the fat body- or cardiomyocyte-specific *Gal4* control and *Gal4*>*UAS-RNAi* knockdown from within the same cross on NFD. Importantly, on HFD, we still observed differential effects on normalized TG levels in the cardiomyocyte-specific, but not in fat body-specific *Gal4*-mediated knockdown, compared to their respective *Gal4* control. These results are not confounded by genetic backgrounds as the genotypes are identical regardless of normal fat or high fat diets. In addition, we used more than one *Gal4* driver to generate each tissue-specific knockdown so as to avoid any *Gal4*-specific genetic background effects.

Interestingly, our results showed that *mtp* silencing in the fat body or in cardiomyocytes (Fig 2) induces similar reduction in normalized TG content as that of ubiquitous *mtp* silencing (Fig 1C and 1D). Several scenarios could explain this phenomenon. One possibility is that the ubiquitous knockdown of *mtp* only partially decreases *mtp* expression in both the fat body and cardiomyocytes, whereas knocking down *mtp* expression using either fat body or cardiomyocyte-specific *Gal4* drivers nearly-abolishes Mtp levels in either tissue. Hence, the addictive effects on normalized TG reduction of partially knocking down *mtp* expression in both the fat body and cardiomyocytes mediated by the ubiquitous *Gal4* drivers could be similar to the sole effect of a complete (or near complete) loss of Mtp in either the fat body or cardiomyocytes. On the other hand, it is also possible that the Lpp derived from the cardiomyocytes and from fat body could be further modified so that each population plays an important but distinct role in the regulation of systemic TG levels. It is also possible that fat body and cardiomyocytes could contribute to Lpp production in a differentially circadian fashion—they generate Lpp at different times of day. Each of the above scenarios does not require the contributions of the cardiomyocytes and fat body to be additive.

Excessive dietary fat consumption has been shown to differentially alter the relative levels of Mtp or apoB in different tissues such as the liver and intestine in various mammalian models [29–35]. However, whether such differential changes in Mtp and apoLpp expression in different tissues in response to HFD could affect overall lipid metabolism is not known. Here, we reveal that in *Drosophila*, high dietary fat also affects Mtp and apoLpp expression differently in different tissues, with HFD dramatically increasing relative Mtp and apoLpp expression levels in the cardiomyocytes and strongly diminishing relative Mtp and apoLpp levels in the fat body, thus culminating in significantly higher levels of Mtp and apoLpp in the cardiomyocytes than in the fat body (Fig 7B). One functional consequence of such, as suggested by our results, could be to modulate the relative input of the apoB-lipoproteins derived from different tissues on systemic lipid homeostasis regulation (Fig 7B). It remains to be determined how HFD could differentially alter Mtp or apoLpp gene expression in the cardiomyocytes versus the fat body, and how the subsequent alteration of apoB-lipoprotein production from the cardiomyocytes versus fat body could impact the pathogenesis of obesity.

In conclusion, our results reveal a novel and significant function of the heart-mediated apoB-lipoproteins in the control of systemic lipid metabolism in *Drosophila*. Given the conservation of apoB-lipoprotein−mediated lipid transport pathways [36], these findings could provide new insights into mammalian apoB-lipoprotein physiology and regulation.

## Materials and Methods

### Fly stocks

The following strains were obtained from the Bloomington Stock Center: *w*^*1118*^ (BL6326), *UAS-mtp*^*RNAi*^ (VDRC15775), *UAS-apoLpp*^*RNAi*^ (BL33388), *Da-Gal4* (BL5460), *Arm-Gal4* (BL1560), *Elav-Gal4* (BL458), *cad-Gal4* (BL3042), *R4-Gal4* (BL33832), *Lsp2-Gal4* (BL6357), *ppl-Gal4* (BL58768), *Mhc-Gal4* (BL38464), *UAS-mCD8-GFP* (BL5137), and the Bloomington deficiency kit for chromosome 2L (DK2L). DK2L contains 102 stocks with molecularly defined genomic deletions generated primarily by two groups, Exelixis and DrosDel [37]. The deficiencies were generated using FLP recombinase mediated excision of genomic DNA flanked by two neighboring *in trans* P-elements (i.e. Piggybac and P(RS3)/P(XP) P-elements) [38–41]. The three deficiency lines further characterized in this study are *Df(2L)ED1378* (BL9682), *Df(2L)BSC333* (BL 24357) and *Df(2L)Exel7080* (BL7853). *UAS-mtp*^*RNAi*^ was obtained from the Vienna Drosophila Resource Center (15775). Other stocks used in this study were as follows: *Sns-Gal4* (a gift of Susan Abmayr, Stowers Institute, Kansas City); *Hand-Gal4* (a gift of Lauren Perrin, TAGC, France); *TinCΔ4-Gal4* (abbreviated here to *TinC-Gal4*) (a gift of Rolf Bodmer, Sanford-Burnham-Presby Medical Discovery Institute, La Jolla). The *mtp* cDNA was obtained from Berkeley *Drosophila* Genome Project-*Drosophila* Genome Collection (BDGP-DGC) clone containing full-length *mtp* (*mtp*^*+*^) cDNA (SD 01501). BDGP-DGC *mtp* clone was cloned into pUAST vector. *UAS-mtp*^*+*^ transgenic flies were generated by Rainbow Transgenics.

### Feeding regimen and genetic screening

Parental flies include control *w*^*1118*^ flies, flies from each of the 102 stocks from the 2L Deficiency kit (DK2L), or for *Gal4/UAS* crosses, flies harboring the appropriate *Gal4* driver with flies harboring *UAS*-*transgene*^*RNAi*^. Parental male and female flies (n = 35–40) were acclimatized on NFD for 1–2 days before being transferred to a fresh NFD vial or a fresh vial containing HFD food. Parents were maintained in their respective food vials until a single uniform layer of eggs was deposited on the surface of food (typically in ~2 days for both NFD- and HFD-fed flies). Parental flies were then removed and the progenies continued to be reared on the same NFD or HFD vial. Progenies were collected at two different developmental windows–third instar larval and newly eclosed adult stages − for various analyses. Foraging third instar larvae were collected for blue food feeding assays (see below). Wandering third instar larvae were collected for the Nile Red staining of neutral lipid, for Western blot or immunofluorescence analysis of Mtp and apoLpp levels in the fat body, heart, or intestine, and for heart function analyses. Newly-eclosed male and female adults (equal ratio) were collected for the measurement of whole-body TG levels and analyses of whole-body phospho-Akt levels.

### Feeding assay

Foraging (feeding) third instar larvae were selected as described previously [42]. Briefly, parental flies as described above were allowed to lay eggs overnight in fly cages containing plates (100 mm x 15 mm, Fisher Scientific) with NFD. The following day, following a 2 hour pre-collection, a 1 hour-egg collection was performed. At 21 hr after egg laying, all hatched larvae were removed from the collection plate under a dissection microscope and the remaining eggs further incubated for 3 hrs (corresponding to 0–3 hr after hatching (AH)). Approximately 30–40 newly hatched larvae were collected and transferred to a fresh NFD plate and allowed to grow until 65–75 hr AH (early and late foraging third instar larvae) before being transferred to NFD or HFD blue food, which was prepared in the same manner as NFD and HFD, except for the addition of 1% (v/v) bromophenol blue (FD&C Blue Dye no. 1; Durkee). Larvae were allowed to feed on the respective blue diets for 1 hour at room temperature, washed 3x in PBS, placed in 1.5 ml tubes and immediately frozen in liquid nitrogen and stored in -80°C. Larvae (n = 5) were subsequently homogenized in 100 μl ice-cold PBS containing 0.05% Triton X-100 (Sigma). Homogenates were centrifuged at 13,000 rpm for 20 min and the supernatants were removed and re-centrifuged at 13,000 rpm for 5 min. The supernatants were transferred to a 96-well plate and the absorbance measured at 630 nm (SpectraMax Plus 384, Molecular Devices, CA).

### Triglyceride assay

Triglyceride assay was performed as previously described [43]. Flies (10–15 per genotype) or third-instar larvae (10–15 per genotype) were weighed and homogenized in PBS containing 0.1% Triton-X100 in an amount (μl) that is 8 X the total weight of flies (μg). The homogenates were centrifuged and the supernatants were removed and incubated with Infinity Triglyceride Reagent (Thermo Electron) for 30 min at 37°C. The absorbance at 540 nm was then measured and TG content was calculated from a standard curve constructed with solutions of known TG concentrations (Thermo Electron). The results were normalized to the protein concentration (μg/μl) of each sample (Bradford assay).

### Nile red lipid staining

The fat bodies, intestines or hearts were dissected from wandering third instar larvae in 5 μg/ml of Nile Red solution and then mounted. Slides were examined with a laser confocal microscope (Leica DMRB) at 40X magnification.

### Western blot analysis

Ten newly eclosed flies per genotype (for phospho-Akt), or fat bodies from twelve wandering third instar larvae per genotype, or heart tubes from seventy wandering third instar larvae per genotype were collected and homogenized in PBS containing 0.1% Triton-X100 and phosphatase and protease inhibitors (Sigma). Samples of 30~40 μg protein were resolved by SDS-PAGE using Novex NuPAGE 10% Bis-Tris Gels (Invitrogen). For western blot analyses of circulating Lpp, hemolymph extraction was performed as described in [44]. Basically, third-instar larvae were thoroughly washed and dried, then placed on a piece of parafilm and pierced with fine forceps. Hemolymph was collected using fine capillary glass micropipettes and diluted 10-fold on ice with phosphate-buffered saline. Protein quantification was performed on the diluted samples and as precisely as possible, twenty-three μg protein per sample were carefully loaded onto each well and resolved by SDS-PAGE using Novex NuPAGE 10% Bis-Tris Gels (Invitrogen). Proteins were transferred to nitrocellulose membranes (Invitrogen). Membranes were incubated overnight at 4°C with the following primary antibodies at the indicated dilutions—Anti-Akt (pan) (rabbit monoclonal 1:1000, Cell Signaling Technology, clone C67E7), anti-pAKT (Ser473, rabbit polyclonal 1:1000, Cell Signaling Technology), anti-Mtp (Microsomal triglyceride transfer protein large subunit) (rabbit polyclonal 1:1000, Aviva Systems Biology), anti-lipophorin (small subunit, rabbit polyclonal 1:20000) [45], anti-tubulin (mouse monoclonal 1:5000, Sigma), anti-β actin (mouse monoclonal 1:1000, GeneTex Inc.). After washing, membranes were incubated with horseradish peroxidase-conjugated anti-mouse and anti-rabbit secondary antibodies (Perkin Elmer) and bands were visualized with ECL^TM^ Western Blotting Detection Reagents (Amersham).

### Immunostaining

Wandering third instar larval intestines were dissected and fixed for 20 minutes with 4% formaldehyde dissolved in PBS at room temperature for apoLpp staining. Wandering third instar larval hearts were dissected on sylgard dishes and fixed for 10 minutes with Bouin’s solution (saturated picric acid:formaldehyde:acetic acid in 15:5:1 ratio) at room temperature for Mtp staining. For apoLpp staining, wandering third instar hearts were dissected on sylguard dishes and fixed either with 4% formaldehyde dissolved in PBS at room temperature or with Bouin’s solution for 10 minutes at room temperature. The fixed tissues were washed for 1 hour in PBS containing 1.0% Triton X-100 (PBT) and incubated overnight at 4°C with the anti-lipophorin antibody (full-length, rabbit polyclonal 1:500) [45] or with the anti-Boca antibody (guinea pig 1:500) [28]. The anti-lipophorin antibodies (full-length and small subunit) as well as anti-Boca antibody were generous gifts from Dr. Joaquim Culi, CSIC-UPO. The tissues were washed three times for a total of 1 hour in PBT and incubated with alexa-fluor-conjugated secondary antibodies (donkey anti-rabbit, 1;150, Jackson Immunoresearch) for 2 hours at room temperature. After washing as before, tissues were mounted in vectashield media and viewed under a laser confocal microscope (Olympus FV1000).

### RT-PCR

For whole fly samples, total RNA was extracted with Trizol reagent (Ambion). cDNA was synthesized from total RNA (1 μg each) with oligo(dT) and superscript III reverse transcriptase (Invitrogen). Subsequently, DNA was amplified using PCR master mix (Invitrogen) and the following oligonucleotides and PCR conditions: 2 min at 95°C, (30 sec at 95°C, 30 sec at 55°C, and 30 sec at 72°C) × 30, and 10 min at 72°C. Actin-5C was used as an internal control. Primers used were as follows:

Actin-5C    F: 5’-GGCGTAATGG TAGGAATGGG ACAAA-3’

                    R: 5’-GTGCTTTCT CTCTACGCCT CCGG-3’

Mtp              F: 5’-TTGCTCGCTTGTGGTTTCCG-3’

                    R: 5’-CCTGTAAGGCAGCCACCGATAAA -3’

### Real-time quantitative PCR (qPCR) analysis

For RNA extraction, 60 hearts from wandering third instar larvae or fat bodies from 20 wandering third instar larvae were dissected and collected in Trizol reagent (Ambion). cDNA using oligo(dT) were synthesized from total RNA (400 ng each) with superscript III reverse transcriptase (Invitrogen). qPCR amplification reactions were performed in triplicate, by mixing 1 μl of RT product with 10 μl of SYBR qPCR Mastermix (Qiagen) containing the appropriate PCR primers. Thermal cycling and florescence monitoring were performed in a CFX96 (Bio-rad) using the following PCR conditions: 6 min at 95°C, 30 sec at 95°C, 30 sec at 60°C, and 30 sec at 72°C) × 35, 15 sec at 95°C, 31 sec at 65°C and 0.5 sec at 65°C. Values were normalized with gapdh. Primers used were as follows:

Gapdh F: 5’- GCGTCACCTGAAGATCCCAT -3’

                R: 5’- GAAGTGGTTCGCCTGGAAGA -3’

Mtp      F: 5’- ACGGAAATCCAGCAGAACACT -3’

                R: 5’- ATACGTAAAGCCAACGGCCA -3’

apoLpp    F: 5’- AATTCGCGGATGGTCTGTGT -3’

                R: 5’- GCCCCTTAGGGATAGCCTTT -3’

### Semi-intact drosophila heart preparation and heartbeat analysis

Cardiac contractility measurements were performed as described previously [46] on semi-quantitative preparations of larval hearts. Briefly, wandering third instar larvae or newly-eclosed adult females of different genotypes (about 20 larvae per genotype) were pinned down in the anterior and posterior regions on a sylgard dissecting dish and carefully dissected to reveal the beating hearts in an artificial hemolymph buffer [47]. High-speed 30-s movies were recorded at a rate of >150 frames per second using a Hamamatsu CCD camera on a Nikon 80i upright microscope with a 10x dipping immersion lens. The images were processed using Simple PCI imaging software (Compix Inc.). M-modes and quantitative data were generated using a MatLab-based image analysis program [46].

### Statistical analysis

All data are presented as the mean ± SEM of the indicated number of replicates. Data were analyzed using the two-tailed Student’s *t*-test and *p* < 0.05 was considered statistically significant.

## Supporting Information

S1 Fig Generation of a high-fat diet-induced model of obesity in *Drosophila*. (A) Experimental workflow for generating obese control *w*^*1118*^ flies and for screening flies from each of the 102 lines from the 2L Deficiency kit (DK2L).
(B, B′) Nutritional composition of NFD (B) and HFD (B′). These diets were of the same (NFD) or similar (HFD) compositions to diets used in similar *Drosophila* studies [48]. Yellow highlights the % contribution of total kcal by fat.
(C) Whole-body TG levels in newly eclosed control *w*^*1118*^ flies (1:1 ratio of males:females) on NFD or HFD. TG levels were normalized to total protein. Results are the mean ± SEM of the indicated number of flies (N) analyzed over at least 4 independent experiments. *P*-values are from Student’s *t*-test.
(D) Representative confocal images of Nile Red-stained lipid droplets in the fat body of control *w*^*1118*^ third instar larvae on NFD or HFD. Arrows indicate lipid droplets and white polygons indicate single fat cells or adipocytes.
(TIF)

S2 Fig Mtp and apoLpp protein content is reduced in the fat body using Mtp RNAi and different fat body-specific *Gal4* drivers. (A, B) Western blot analysis of Mtp in the fat body of third instar larvae with *Gal4* driver only. *R4-Gal4* (A) or *ppl-Gal4* (B) and larvae with fat body-specific knockdown of *mtp* using *R4-Gal4* (A) or *ppl-Gal4* (B) on NFD. α-Tubulin was used as loading control. About thirty μg of protein was loaded per lane.
(C) Representative confocal images of apoLpp staining in the hearts of control third instar larvae (*Hand-Gal4*, left) and third instar larvae with heart-specific knockdown of *apoLpp* using *Hand-Gal4* (right) on HFD. Scale bars represent 20 μm. Arrows indicate the apoLpp puncta.
(TIF)

S3 Fig Systemic lipid levels under normal food diet and high fat diet conditions in wandering third instar larvae with inhibition of Mtp or apoLpp in the fat body or cardiomyocytes. (A) Whole-body TG levels of wandering third instar larvae with *Gal4* drivers only (controls) or with fat body-specific knockdown of *mtp* using *ppl-Gal4* on NFD (blue) or HFD (red). *P*-values are from Student’s *t*-test and are between *Gal4* control and *Gal4*-mediated RNAi lines within NFD or HFD.
(B, C) Whole-body TG levels of wandering third instar larvae with *Gal4* drivers only (controls) or with cardiomyocyte-specific knockdown of *mtp* using *Hand-Gal4* (B), or *TinC-Gal4* (C) on NFD (blue) or HFD (red). *P*-values are from Student’s *t*-test and are between *Gal4* control and *Gal4*-mediated RNAi lines within NFD or HFD. In all cases, TG levels (μg/μl) were normalized to total protein (μg/μl). Results are expressed as the fold change in whole larval normalized TG compared with that of the control larvae (set to 1.0 for NFD or HFD). Results are the mean ± SEM of the indicated number of larvae (N) analyzed over at least 5 independent experiments.
(TIF)

S4 Fig Mtp knockdown in the pericardial cell, neuronal cell or enterocyte does not affect systemic lipid metabolism on normal food diet and high fat diet. (A-C) Whole-body TG levels of newly-eclosed flies with *Gal4* drivers only (controls) or with tissue-specific knockdown of *mtp* using pericardial cell-specific driver *Sns-Gal4* (A), neuronal-specific driver *ElaV-Gal4* (B), or intestinal-specific driver *cad-Gal4* (C) on NFD (blue) or HFD (red). In all cases, TG levels (μg/μl) were normalized to total protein (μg/μl). Results are expressed as the fold change in whole fly normalized TG compared with that of the control flies on NFD (set to 1.0). Results are the mean ± SEM of the indicated number of flies (N) analyzed over at least 5 independent experiments. *P*-values are from Student’s *t*-test and are between *Gal4* control and *Gal4*-mediated RNAi lines within NFD or HFD.
(TIF)

S5 Fig Larval heart function is not affected by high fat diet intake. (A, A’) Representative confocal images of Nile Red-stained lipid droplets in the hearts of control (*w*^*1118*^) third instar larvae on NFD and HFD. Arrows indicate lipid droplets.
(B) Representative M-mode traces (5 s) showing movement of heart tube walls (Y-axis) versus time (X-axis) for hearts of control (*w*^*1118*^) third instar larvae on NFD and HFD.
(C) Heart periods of control (*w*^*1118*^) third instar larvae on NFD and HFD. Results are the mean ± SEM of the indicated number of larvae (N).
(D) Arrhythmia index (AI) of control (*w*^*1118*^) third instar larvae on NFD and HFD. Results are the mean ± SEM of the indicated number of larvae (N).
(E) Cardiac contractility changes (measured as % fractional shortening) of control (*w*^*1118*^) third instar larvae on NFD and HFD. Results are the mean ± SEM of the indicated number of larvae (N).
(F) Diastolic diameter (diameter during relaxation) of the heart tubes in control (*w*^*1118*^) third instar larvae on NFD and HFD. Results are the mean ± SEM of the indicated number of larvae (N).
(G) Systolic diameter (diameter during contraction) of the heart tubes in control (*w*^*1118*^) third instar larvae on NFD and HFD. Results are the mean ± SEM of the indicated number of larvae (N). In all cases, *P*-values are from Student’s *t*-test and are between control *w*^*1118*^ lines on NFD and HFD.
(TIF)

S6 Fig Adult heart function is not affected by Mtp inhibition in the heart on normal food diet or high fat diet. (A) Heart periods of newly-eclosed female flies with *Gal4* drivers only (*Hand-Gal4*) or with cardiomyocyte-specific knockdown of *mtp* using *Hand-Gal4* on NFD and HFD.
(B) Arrhythmia index (AI) of newly-eclosed female flies with *Gal4* drivers only (*Hand-Gal4*) or with cardiomyocyte-specific knockdown of *mtp* using *Hand-Gal4* on NFD and HFD.
(C) Diastolic diameter (diameter during relaxation) of the heart tubes in newly-eclosed female flies with *Gal4* drivers only (*Hand-Gal4*) or with cardiomyocyte-specific knockdown of *mtp* using *Hand-Gal4* on NFD and HFD.
(D) Systolic diameter (diameter during contraction) of the heart tubes in newly-eclosed female flies with *Gal4* drivers only (*Hand-Gal4*) or with cardiomyocyte-specific knockdown of *mtp* using *Hand-Gal4* on NFD and HFD.
(E) Cardiac contractility changes (measured as % fractional shortening) of newly-eclosed female flies with *Gal4* drivers only (*Hand-Gal4*) or with cardiomyocyte-specific knockdown of *mtp* using *Hand-Gal4* on NFD and HFD. In all cases, results are the mean ± SEM of the indicated number of flies (N). *P*-values are from Student’s *t*-test and are between *Gal4* control and *Gal4*-mediated RNAi lines within NFD or HFD.
(TIF)

S7 Fig Inhibition of Mtp only in cardiomyocytes reduces the levels of intestinal Lpp on high fat diet. (A-C) Representative confocal images of apoLpp staining in the intestines of control third instar larvae (*Hand-Gal4*, left), in the intestines of third instar larvae with heart-specific knockdown of *mtp* using *Hand-Gal4* (middle), and in the intestines of third instar larvae with heart-specific knockdown of *apoLpp* using *Hand-Gal4* (right) on HFD. In all cases, arrows indicate the apoLpp puncta which reflect the presence of Lpp. Scale bars represent 40 μm.
(TIF)

S8 Fig High fat diet alters relative Mtp and apoLpp protein levels in the fat body. (A) Western blot analysis of Mtp protein level in the fat body of control (*w*^*1118*^) third instar larvae on NFD and HFD. α-Tubulin was used as loading control. Forty μg of protein were loaded per lane.
(B) Western blot analysis of apoLpp protein levels in the fat body of control (*w*^*1118*^) third instar larvae on NFD and HFD. α-Tubulin was used as loading control. Twenty μg of protein were loaded per lane.
(TIF)

S9 Fig Inhibition of Mtp or apoLpp in the fat body or cardiomyocytes differentially alters circulating Lpp abundance under NFD and HFD conditions. (A, B) Western blot analysis of apoLpp in the hemolymph extracted from third instar larvae with *Gal4* driver only (*ppl-Gal4*) or from third instar larvae with fat body-specific knockdown of *mtp* using *ppl-Gal4* on NFD (A) and HFD (B). 1 μl of extracted hemolymph was diluted 10-fold and 5 μl of the diluted sample used for Bradford protein assay. Based on the protein assay, twenty-three μg of protein were loaded per lane.
(C, D) Western blot analysis of apoLpp in the hemolymph extracted from third instar larvae with *Gal4* driver only (*Hand-Gal4*) or from third instar larvae with fat body-specific knockdown of *mtp* using *Hand-Gal4* on NFD (C) and HFD (D). 1 μl of extracted hemolymph was diluted 10-fold and 5 μl of the diluted sample used for Bradford protein assay. Based on the protein assay, twenty-three μg of protein were loaded per lane.
(TIF)

S1 Table Effects of *mtp* or *apoLppi* knockdown mediated by all drivers used in this study on developmental timing and viability. (TIF)
