# Supplementary figures and images for: Cardiomyocyte Regulation of Systemic Lipid Metabolism by the Apolipoprotein B-Containing Lipoproteins in Drosophila
Source: PLoS Genet. 2017 Jan 17;13(1):e1006555. doi: 10.1371/journal.pgen.1006555 (PMC5283750; doi:10.1371/journal.pgen.1006555)

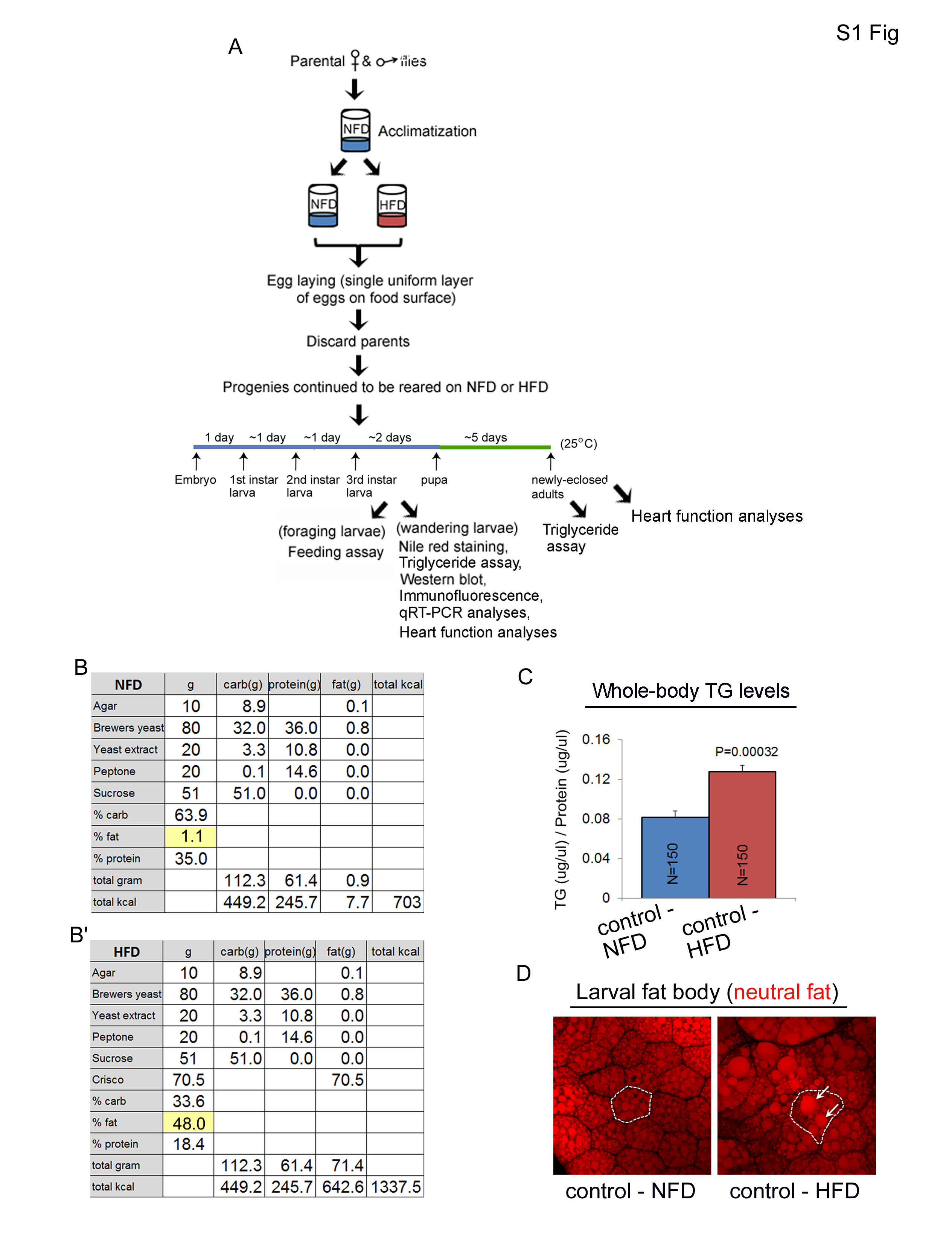

Supplement: S1 Fig — (A) Experimental workflow for generating obese control w1118 flies and for screening flies from each of the 102 lines from the 2L Deficiency kit (DK2L). (B, B′) Nutritional composition of NFD (B) and HFD (B′). These diets were of the same (NFD) or similar (HFD) compositions to diets used in similar Drosophila studies [48]. Yellow highlights the % contribution of total kcal by fat. (C) Whole-body TG levels in newly eclosed control w1118 flies (1:1 ratio of males:females) on NFD or HFD. TG levels were normalized to total protein. Results are the mean ± SEM of the indicated number of flies (N) analyzed over at least 4 independent experiments. P-values are from Student’s t-test. (D) Representative confocal images of Nile Red-stained lipid droplets in the fat body of control w1118 third instar larvae on NFD or HFD. Arrows indicate lipid droplets and white polygons indicate single fat cells or adipocytes. (TIF) [file pgen.1006555.s001.tif]

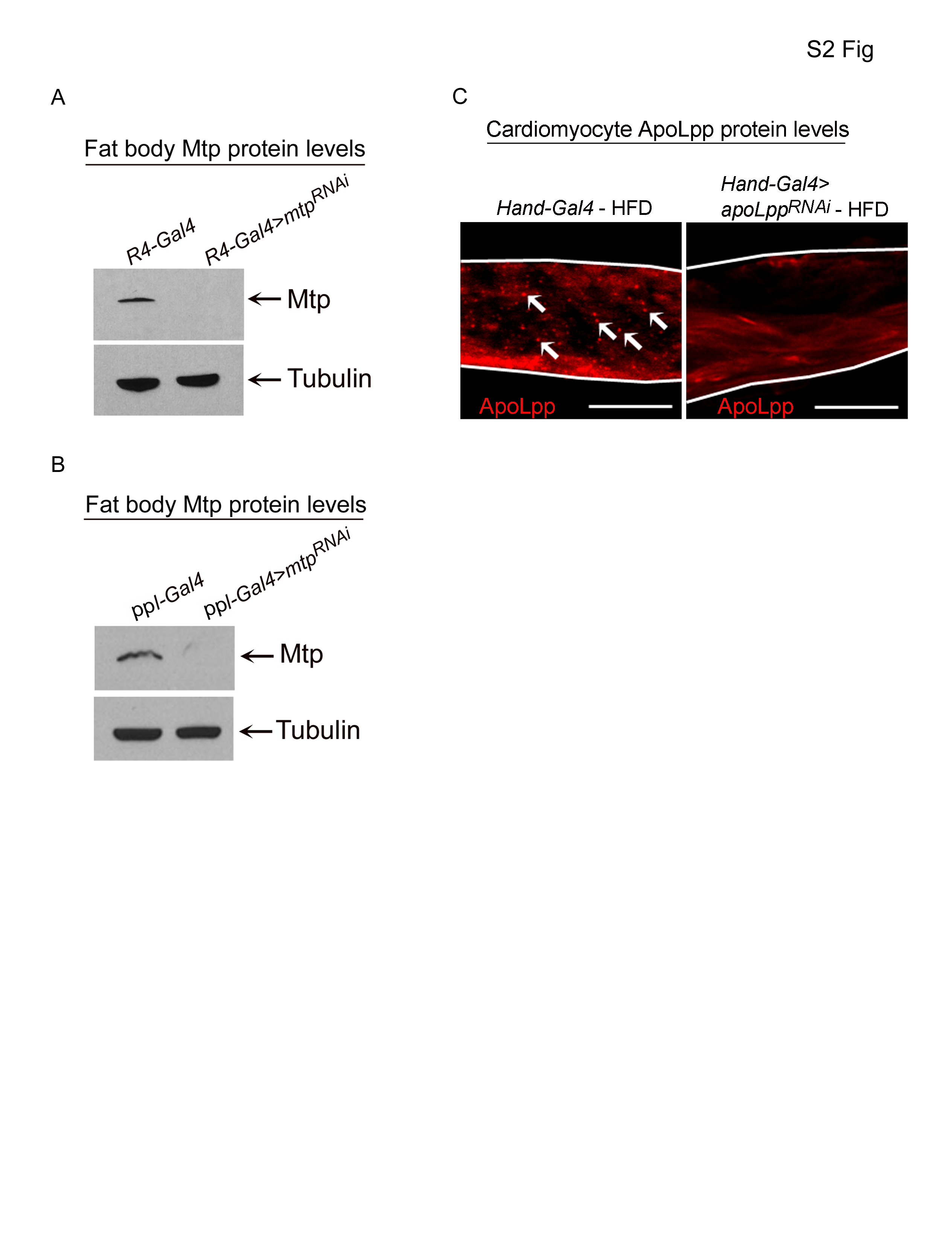

Supplement: S2 Fig — (A, B) Western blot analysis of Mtp in the fat body of third instar larvae with Gal4 driver only. R4-Gal4 (A) or ppl-Gal4 (B) and larvae with fat body-specific knockdown of mtp using R4-Gal4 (A) or ppl-Gal4 (B) on NFD. α-Tubulin was used as loading control. About thirty μg of protein was loaded per lane. (C) Representative confocal images of apoLpp staining in the hearts of control third instar larvae (Hand-Gal4, left) and third instar larvae with heart-specific knockdown of apoLpp using Hand-Gal4 (right) on HFD. Scale bars represent 20 μm. Arrows indicate the apoLpp puncta. (TIF) [file pgen.1006555.s002.tif]

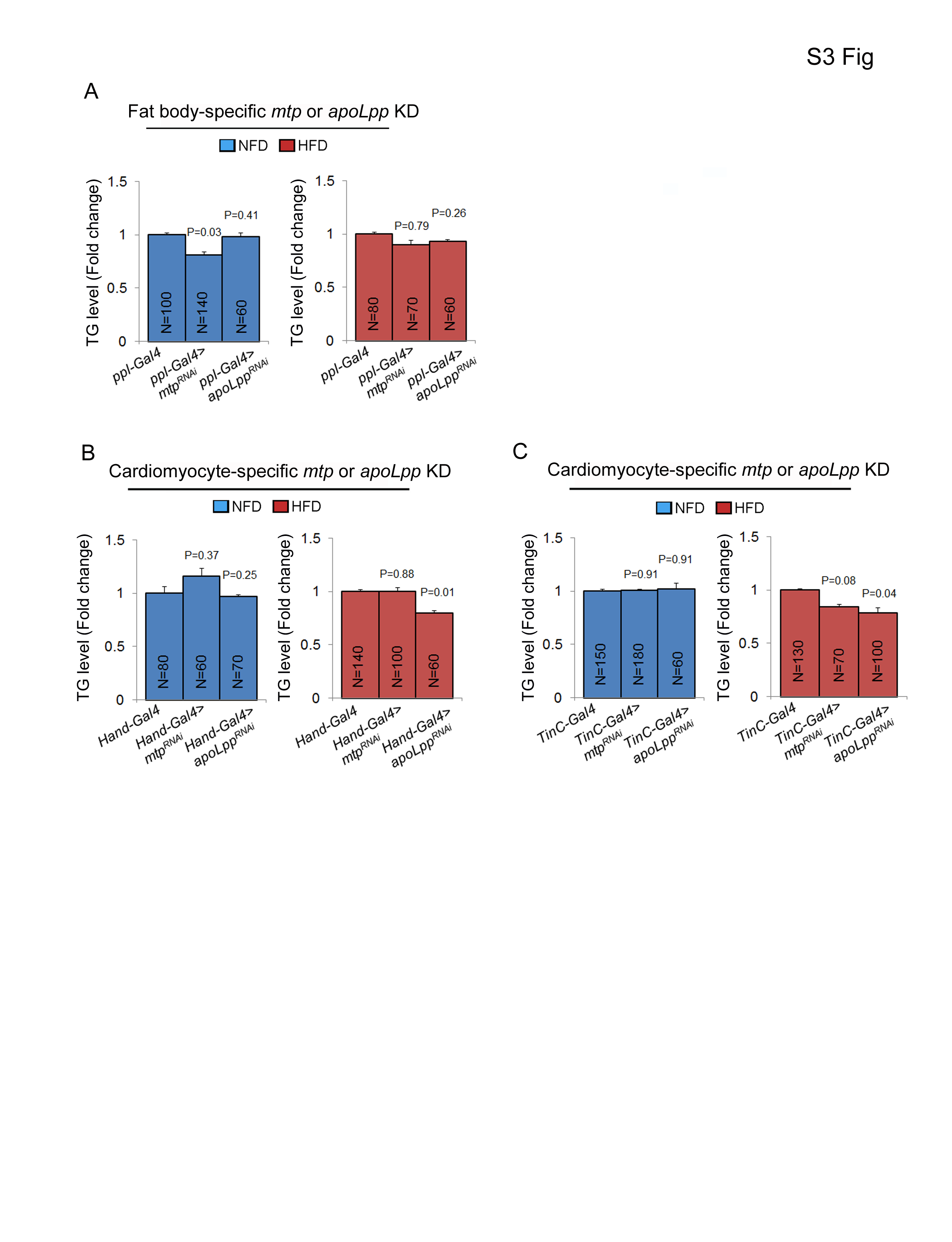

Supplement: S3 Fig — (A) Whole-body TG levels of wandering third instar larvae with Gal4 drivers only (controls) or with fat body-specific knockdown of mtp using ppl-Gal4 on NFD (blue) or HFD (red). P-values are from Student’s t-test and are between Gal4 control and Gal4-mediated RNAi lines within NFD or HFD. (B, C) Whole-body TG levels of wandering third instar larvae with Gal4 drivers only (controls) or with cardiomyocyte-specific knockdown of mtp using Hand-Gal4 (B), or TinC-Gal4 (C) on NFD (blue) or HFD (red). P-values are from Student’s t-test and are between Gal4 control and Gal4-mediated RNAi lines within NFD or HFD. In all cases, TG levels (μg/μl) were normalized to total protein (μg/μl). Results are expressed as the fold change in whole larval normalized TG compared with that of the control larvae (set to 1.0 for NFD or HFD). Results are the mean ± SEM of the indicated number of larvae (N) analyzed over at least 5 independent experiments. (TIF) [file pgen.1006555.s003.tif]

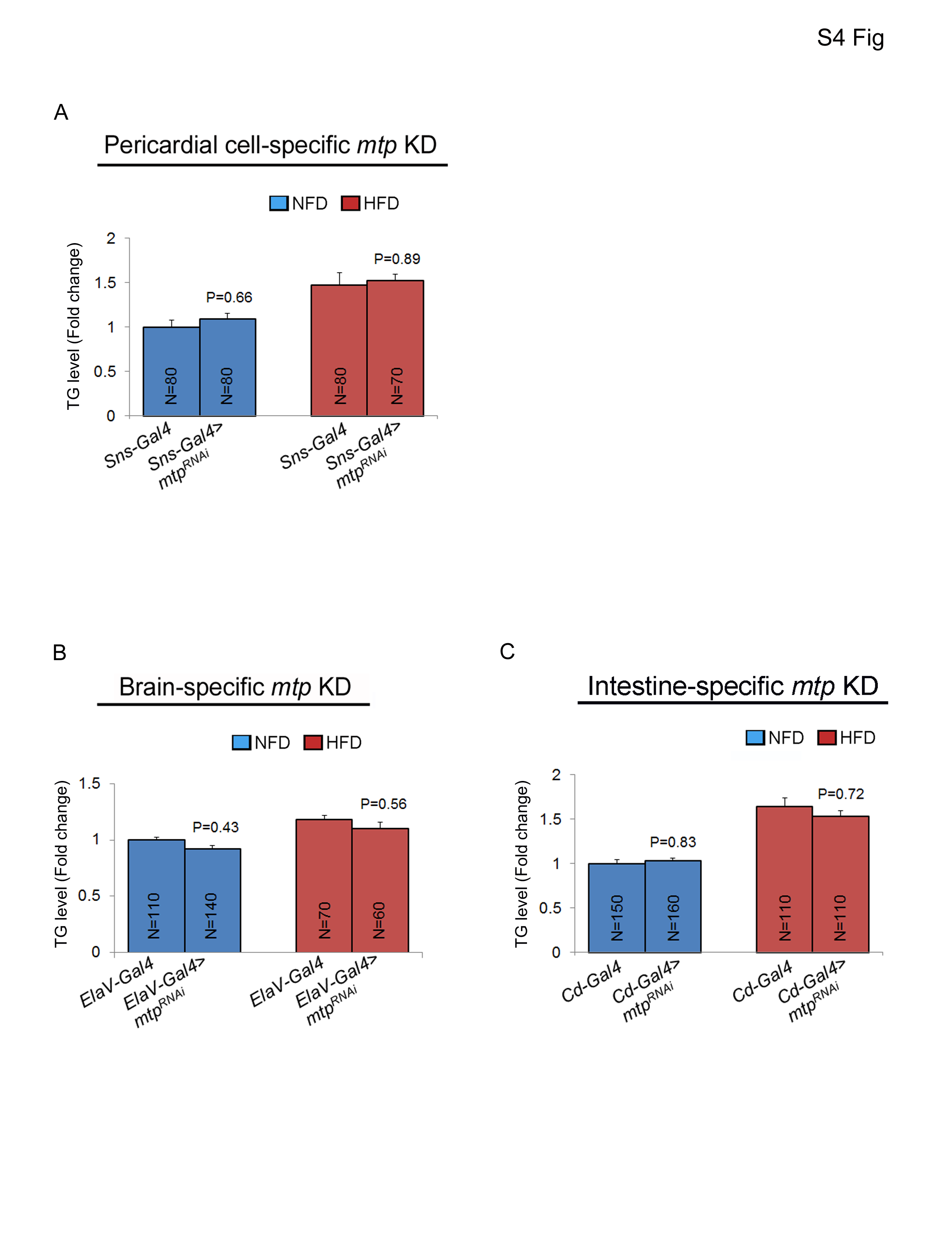

Supplement: S4 Fig — (A-C) Whole-body TG levels of newly-eclosed flies with Gal4 drivers only (controls) or with tissue-specific knockdown of mtp using pericardial cell-specific driver Sns-Gal4 (A), neuronal-specific driver ElaV-Gal4 (B), or intestinal-specific driver cad-Gal4 (C) on NFD (blue) or HFD (red). In all cases, TG levels (μg/μl) were normalized to total protein (μg/μl). Results are expressed as the fold change in whole fly normalized TG compared with that of the control flies on NFD (set to 1.0). Results are the mean ± SEM of the indicated number of flies (N) analyzed over at least 5 independent experiments. P-values are from Student’s t-test and are between Gal4 control and Gal4-mediated RNAi lines within NFD or HFD. (TIF) [file pgen.1006555.s004.tif]

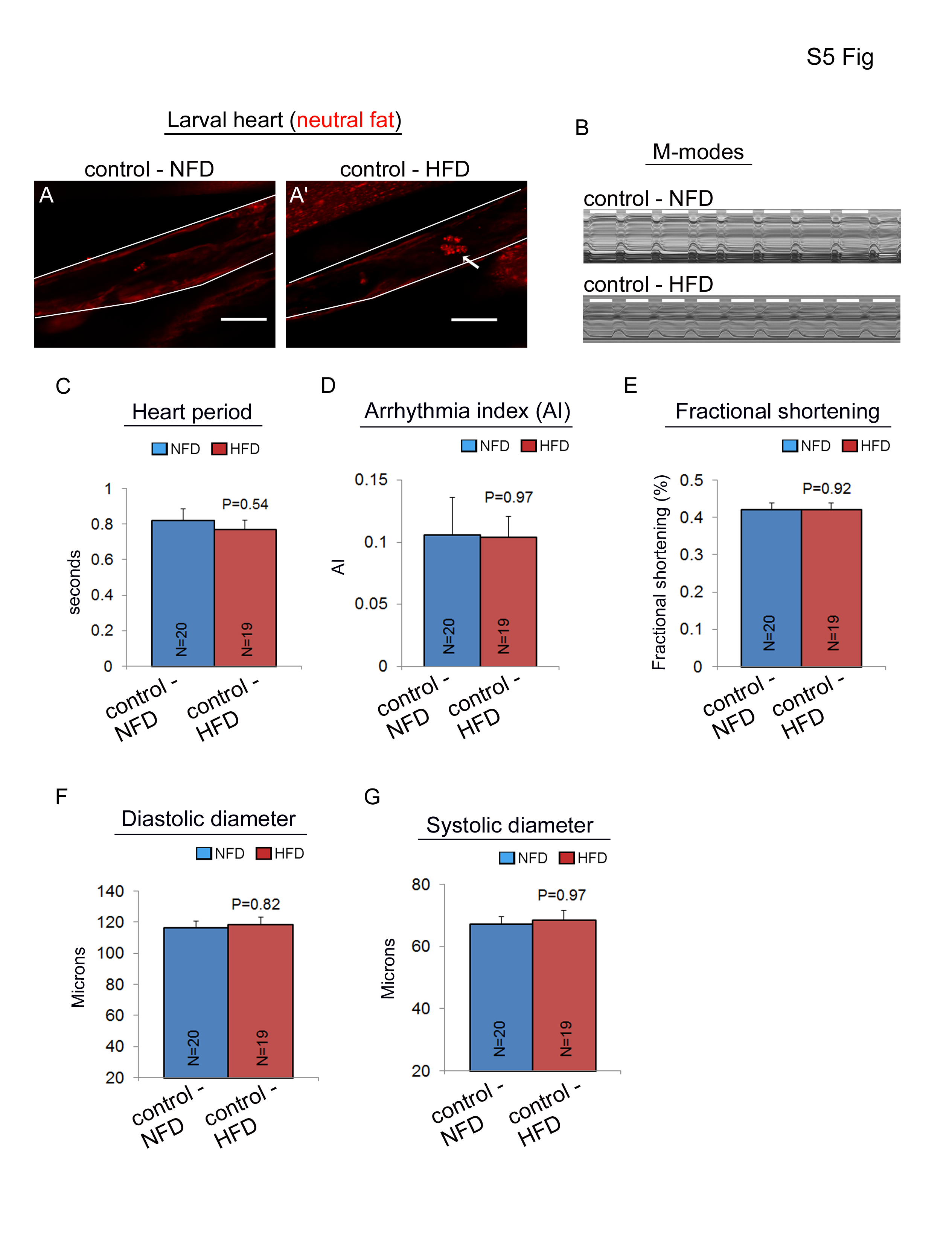

Supplement: S5 Fig — (A, A’) Representative confocal images of Nile Red-stained lipid droplets in the hearts of control (w1118) third instar larvae on NFD and HFD. Arrows indicate lipid droplets. (B) Representative M-mode traces (5 s) showing movement of heart tube walls (Y-axis) versus time (X-axis) for hearts of control (w1118) third instar larvae on NFD and HFD. (C) Heart periods of control (w1118) third instar larvae on NFD and HFD. Results are the mean ± SEM of the indicated number of larvae (N). (D) Arrhythmia index (AI) of control (w1118) third instar larvae on NFD and HFD. Results are the mean ± SEM of the indicated number of larvae (N). (E) Cardiac contractility changes (measured as % fractional shortening) of control (w1118) third instar larvae on NFD and HFD. Results are the mean ± SEM of the indicated number of larvae (N). (F) Diastolic diameter (diameter during relaxation) of the heart tubes in control (w1118) third instar larvae on NFD and HFD. Results are the mean ± SEM of the indicated number of larvae (N). (G) Systolic diameter (diameter during contraction) of the heart tubes in control (w1118) third instar larvae on NFD and HFD. Results are the mean ± SEM of the indicated number of larvae (N). In all cases, P-values are from Student’s t-test and are between control w1118 lines on NFD and HFD. (TIF) [file pgen.1006555.s005.tif]

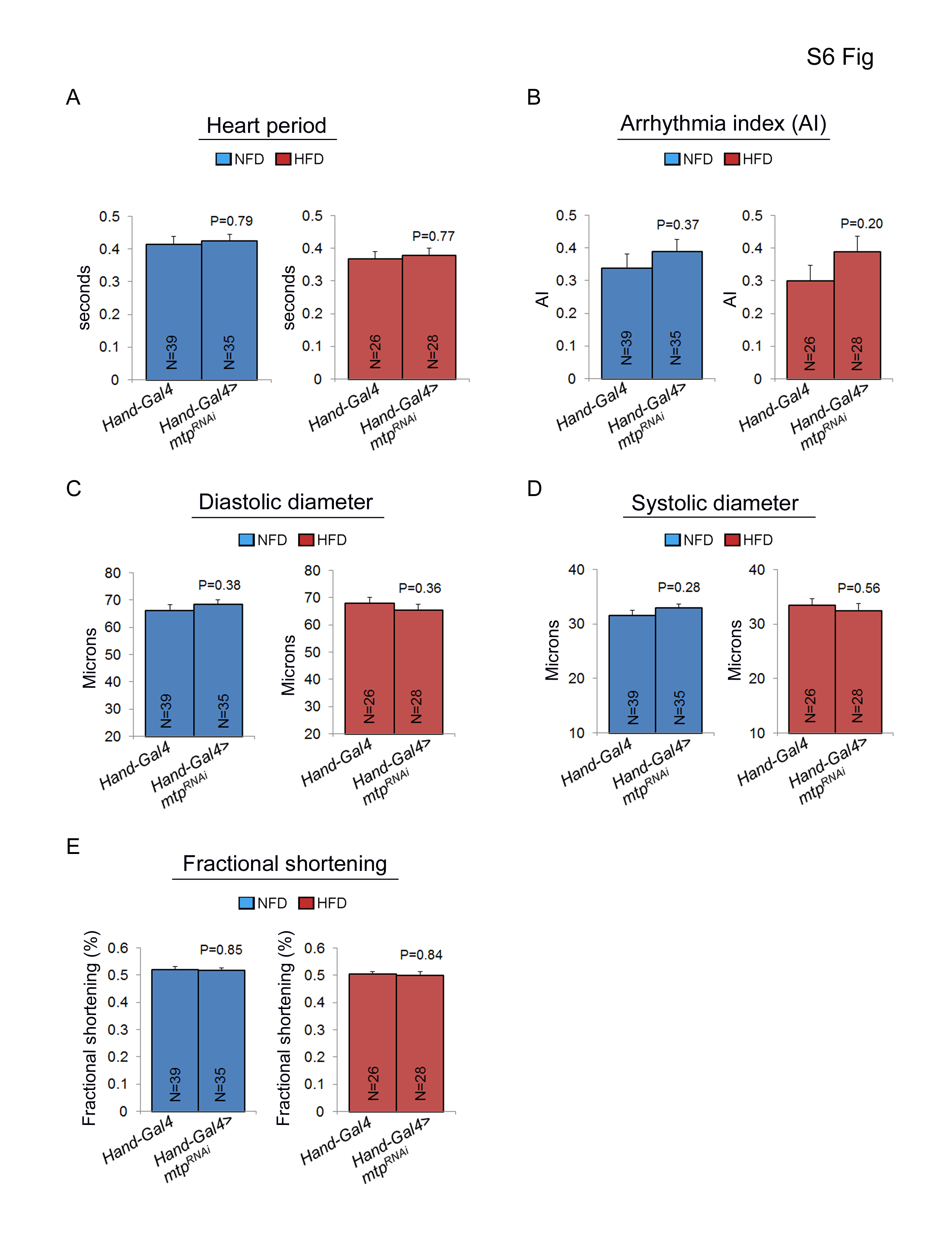

Supplement: S6 Fig — (A) Heart periods of newly-eclosed female flies with Gal4 drivers only (Hand-Gal4) or with cardiomyocyte-specific knockdown of mtp using Hand-Gal4 on NFD and HFD. (B) Arrhythmia index (AI) of newly-eclosed female flies with Gal4 drivers only (Hand-Gal4) or with cardiomyocyte-specific knockdown of mtp using Hand-Gal4 on NFD and HFD. (C) Diastolic diameter (diameter during relaxation) of the heart tubes in newly-eclosed female flies with Gal4 drivers only (Hand-Gal4) or with cardiomyocyte-specific knockdown of mtp using Hand-Gal4 on NFD and HFD. (D) Systolic diameter (diameter during contraction) of the heart tubes in newly-eclosed female flies with Gal4 drivers only (Hand-Gal4) or with cardiomyocyte-specific knockdown of mtp using Hand-Gal4 on NFD and HFD. (E) Cardiac contractility changes (measured as % fractional shortening) of newly-eclosed female flies with Gal4 drivers only (Hand-Gal4) or with cardiomyocyte-specific knockdown of mtp using Hand-Gal4 on NFD and HFD. In all cases, results are the mean ± SEM of the indicated number of flies (N). P-values are from Student’s t-test and are between Gal4 control and Gal4-mediated RNAi lines within NFD or HFD. (TIF) [file pgen.1006555.s006.tif]

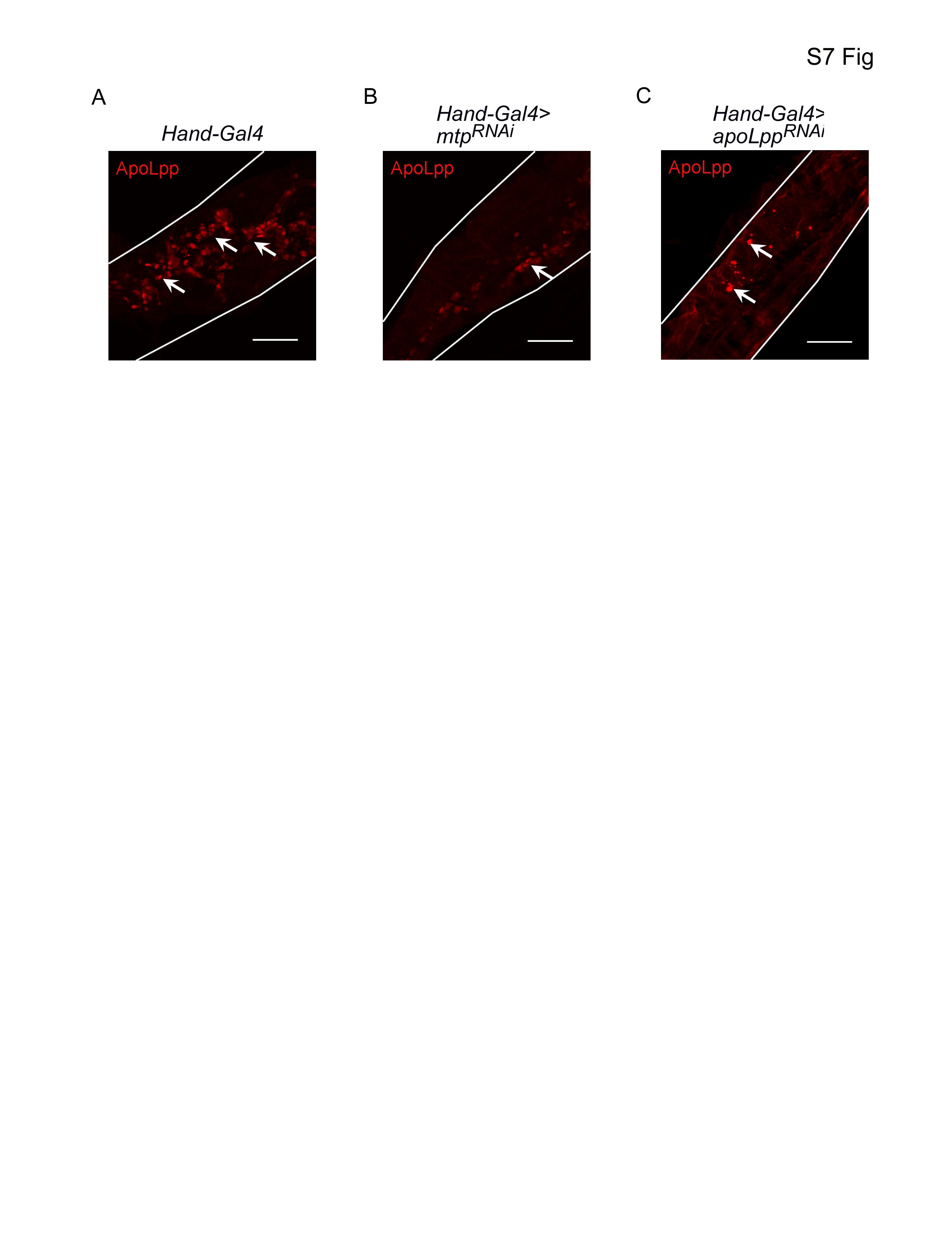

Supplement: S7 Fig — (A-C) Representative confocal images of apoLpp staining in the intestines of control third instar larvae (Hand-Gal4, left), in the intestines of third instar larvae with heart-specific knockdown of mtp using Hand-Gal4 (middle), and in the intestines of third instar larvae with heart-specific knockdown of apoLpp using Hand-Gal4 (right) on HFD. In all cases, arrows indicate the apoLpp puncta which reflect the presence of Lpp. Scale bars represent 40 μm. (TIF) [file pgen.1006555.s007.tif]

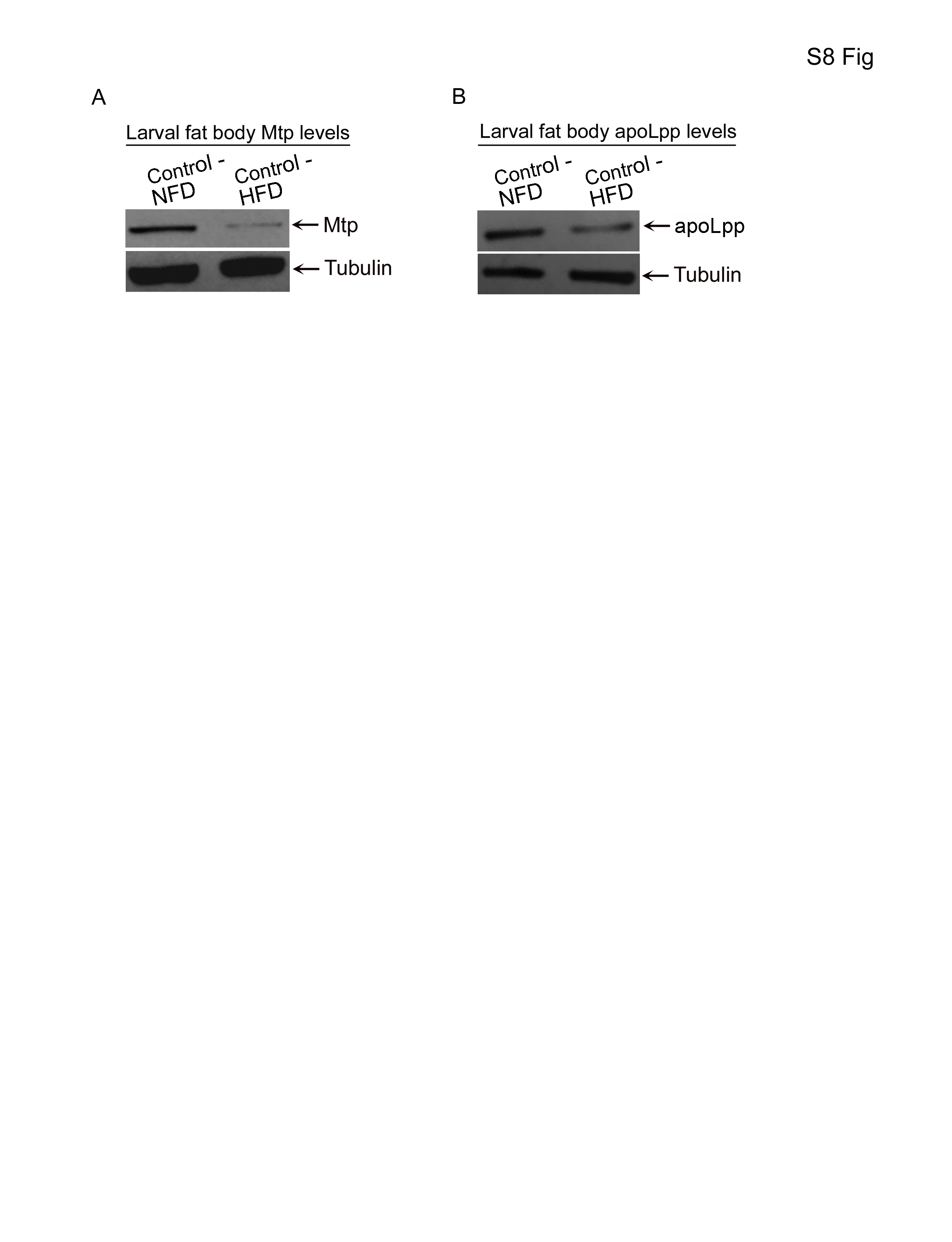

Supplement: S8 Fig — (A) Western blot analysis of Mtp protein level in the fat body of control (w1118) third instar larvae on NFD and HFD. α-Tubulin was used as loading control. Forty μg of protein were loaded per lane. (B) Western blot analysis of apoLpp protein levels in the fat body of control (w1118) third instar larvae on NFD and HFD. α-Tubulin was used as loading control. Twenty μg of protein were loaded per lane. (TIF) [file pgen.1006555.s008.tif]

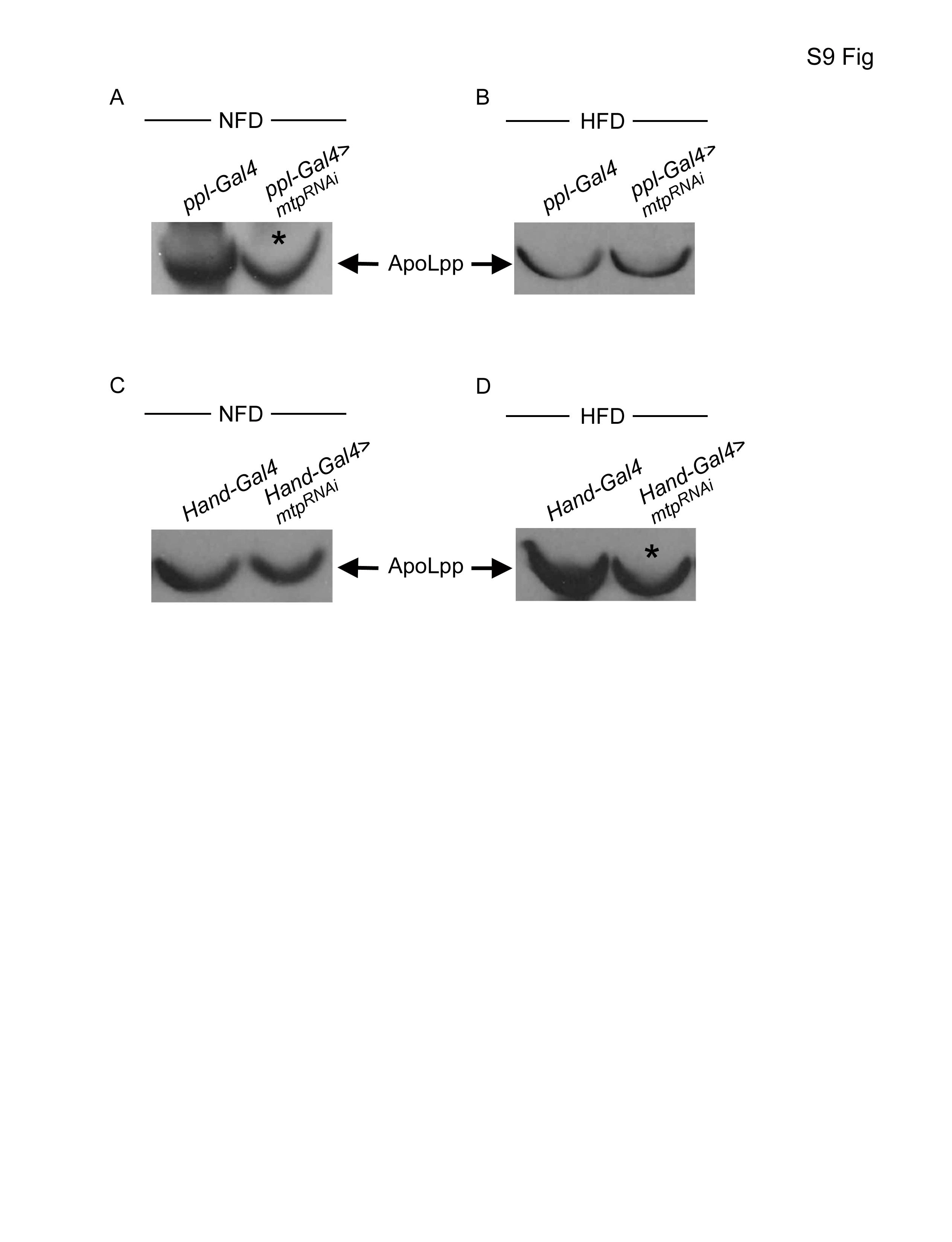

Supplement: S9 Fig — (A, B) Western blot analysis of apoLpp in the hemolymph extracted from third instar larvae with Gal4 driver only (ppl-Gal4) or from third instar larvae with fat body-specific knockdown of mtp using ppl-Gal4 on NFD (A) and HFD (B). 1 μl of extracted hemolymph was diluted 10-fold and 5 μl of the diluted sample used for Bradford protein assay. Based on the protein assay, twenty-three μg of protein were loaded per lane. (C, D) Western blot analysis of apoLpp in the hemolymph extracted from third instar larvae with Gal4 driver only (Hand-Gal4) or from third instar larvae with fat body-specific knockdown of mtp using Hand-Gal4 on NFD (C) and HFD (D). 1 μl of extracted hemolymph was diluted 10-fold and 5 μl of the diluted sample used for Bradford protein assay. Based on the protein assay, twenty-three μg of protein were loaded per lane. (TIF) [file pgen.1006555.s009.tif]

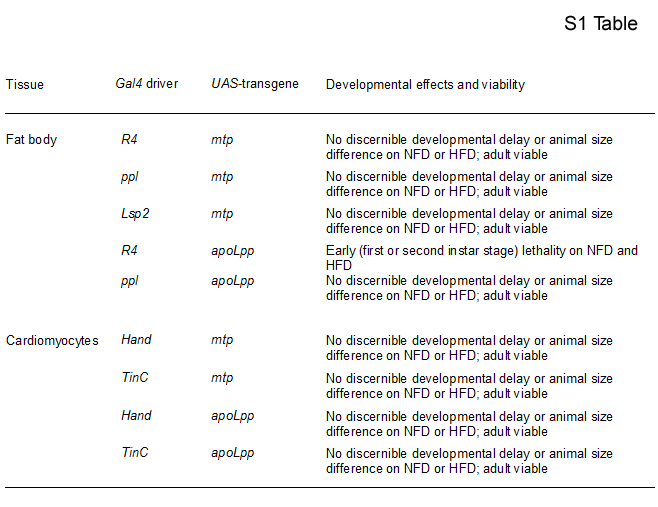

Supplement: S1 Table — (TIF) [file pgen.1006555.s010.tif]
